# A novel multifunctional radioprotective strategy using P7C3 as a countermeasure against ionizing radiation-induced bone loss

**DOI:** 10.1038/s41413-023-00273-w

**Published:** 2023-06-29

**Authors:** Fei Wei, Zewen Kelvin Tuong, Mahmoud Omer, Christopher Ngo, Jackson Asiatico, Michael Kinzel, Abinaya Sindu Pugazhendhi, Annette R. Khaled, Ranajay Ghosh, Melanie Coathup

**Affiliations:** 1 Biionix Cluster, and Department of Internal Medicine, College of Medicine, University of Central Florida, Orlando, FL USA; 2 Molecular Immunity Unit, Department of Medicine, University of Cambridge, Cambridge, UK; 3 Cellular Genetics, Wellcome Sanger Institute, Hinxton, UK; 4 Department of Mechanical and Aerospace Engineering, University of Central Florida, Orlando, FL USA; 5 Burnett School of Biomedical Sciences, College of Medicine, University of Central Florida, Orlando, FL USA

**Keywords:** Bone cancer, Bone cancer

## Abstract

Radiotherapy is a critical component of cancer care but can cause osteoporosis and pathological insufficiency fractures in surrounding and otherwise healthy bone. Presently, no effective countermeasure exists, and ionizing radiation-induced bone damage continues to be a substantial source of pain and morbidity. The purpose of this study was to investigate a small molecule aminopropyl carbazole named P7C3 as a novel radioprotective strategy. Our studies revealed that P7C3 repressed ionizing radiation (IR)-induced osteoclastic activity, inhibited adipogenesis, and promoted osteoblastogenesis and mineral deposition in vitro. We also demonstrated that rodents exposed to clinically equivalent hypofractionated levels of IR in vivo develop weakened, osteoporotic bone. However, the administration of P7C3 significantly inhibited osteoclastic activity, lipid formation and bone marrow adiposity and mitigated tissue loss such that bone maintained its area, architecture, and mechanical strength. Our findings revealed significant enhancement of cellular macromolecule metabolic processes, myeloid cell differentiation, and the proteins LRP-4, TAGLN, ILK, and Tollip, with downregulation of GDF-3, SH2B1, and CD200. These proteins are key in favoring osteoblast over adipogenic progenitor differentiation, cell matrix interactions, and shape and motility, facilitating inflammatory resolution, and suppressing osteoclastogenesis, potentially via Wnt/β-catenin signaling. A concern was whether P7C3 afforded similar protection to cancer cells. Preliminarily, and remarkably, at the same protective P7C3 dose, a significant reduction in triple-negative breast cancer and osteosarcoma cell metabolic activity was found in vitro. Together, these results indicate that P7C3 is a previously undiscovered key regulator of adipo-osteogenic progenitor lineage commitment and may serve as a novel multifunctional therapeutic strategy, leaving IR an effective clinical tool while diminishing the risk of adverse post-IR complications. Our data uncover a new approach for the prevention of radiation-induced bone damage, and further work is needed to investigate its ability to selectively drive cancer cell death.

## Introduction

Cancer remains a leading cause of death worldwide. A combination of surgical resection, radiotherapy, and chemotherapy is commonly used in the treatment of various local and metastasizing cancer cells.^1^ One-half to nearly two-thirds of cancer patients will be exposed to controlled, radiotherapeutic levels of ionizing radiation (IR) at some point during their care.^2–4^ Although IR is a fundamental and necessary tool, exposure of adjacent noncancerous tissues to IR is inevitable and can lead to major morphological and functional damage to otherwise healthy tissue. Due to its high calcium content, bone is estimated to absorb 30%–40% more IR than other tissues, making it a common site for serious ancillary tissue damage.^5^ Osteoradionecrosis, osteoporosis, pathologic insufficiency fractures, and subsequent fracture nonunions are significant complications associated with radiotherapy in cancer survivors, even with fractionation of treatments.^6–11^ As such, the burden of IR-induced damage to healthy bone is a persistent and substantial source of functional impairment, pain, disability, and morbidity.^5,7,9,10,12–14^ The inactive prodrug amifostine has been used as the radioprotector of choice for over the last six decades.^15–17^ Despite progress made to improve the effectiveness of this treatment, none of the strategies have resolved the issue of its side effects. Thus, the FDA approved it for limited clinical indications but not for general clinical use.^17^ Exogenous antioxidants,^18^ such as melatonin,^19^ antiresorptive bisphosphonate treatment, and denosumab (a receptor activator of NF-κB ligand (RANKL) inhibitor), are clinically available;^20^ however, none are capable of completely preventing IR-induced damage, and their long-term efficacy is limited. Thus, no effective preventative countermeasure exists. This issue is important, as the global burden of cancer is expected to increase from 19.3 million new cancer diagnoses and 10 million cancer-related deaths recorded in 2020^21^ to 27.5 million diagnoses and 16.3 million deaths by 2040.^22^ This increase is largely attributed to the increase in our aging (≥65 years) population, together with increased industrial pollution.^23,24^ Thus, a substantial increase in the number of patients to be exposed to radiotherapy is predicted, and the accompanying IR-induced skeletal complications will undoubtedly increase the number and duration of inpatient stays, emergency department visits, and outpatient visits. The exact pathogenesis of IR-induced bone toxicity has not been discovered,^25,26^ and new mechanistic insights combined with an effective countermeasure would be of considerable clinical and therapeutic importance. Therefore, novel and improved therapeutic strategies are urgently needed.

Studies have demonstrated increased IR-induced cell cycle arrest,^27^ inability to proliferate and differentiate,^28,29^ and apoptosis^30^ of bone-forming cells. Recent evidence also indicates that oxidative stress caused by excessive reactive oxygen species (ROS),^31^ macrophage activation and infiltration, and the prolonged release of proinflammatory mediators play an important role in the progression of osteoporosis following IR.^9,32^ Molecularly, and during healthy bone turnover, interactions among the transmembrane receptor activator of NF-κB (RANK), the RANK ligand (RANKL), and the secreted decoy receptor osteoprotegerin are critical in regulating the differentiation and maintenance of osteoclastic activity and the subsequent resorption of bone.^33^ Following IR, the levels of pro-osteoclastic markers (e.g., RANKL) and proadipogenic markers (e.g., peroxisome proliferator activated receptor gamma (PPARγ)) increase, while osteogenic mediators (e.g., alkaline phosphatase (ALP)) decrease.^34^ As a result, excessive bone resorption, combined with impaired bone formation, together with compositional alterations in the adipogenic bone marrow environment, subsequently disrupts cell function and reduces bone quality and strength.

Nicotinamide adenine dinucleotide (NAD) and its reduced form NADH are essential components in various ubiquitous cellular processes, including cell metabolism,^35^ senescence, and individual aging.^36^ NAD exists in an oxidized (NAD^+^) and reduced (NADH) form. In metabolism, NAD^+^ acts as an electron-carrying cofactor for oxidation‒reduction reactions, and electron transfer is the main function of NAD.^37^ As such, NAD^+^/NADH is an important cellular redox pair, among others, and may regulate ROS generation and scavenging.^38^ However, this molecule is also used in other cellular processes, adding or removing chemical groups to or from, for example, proteins. Importantly, cellular levels of NAD^+^ decline significantly during aging,^39^ and decreased NAD^+^ contributes to the loss of osteoprogenitors and bone mass in age-related osteoporosis.^40^ Furthermore, IR therapy causes a significant depletion in NAD^+^ metabolism and disrupts the processes regulating NAD^+^ production and consumption.^41,42^ Pool 7, Compound 3 (P7C3), is an antiapoptotic aminopropyl carbazole with high oral bioavailability that exerts its activity through activation of the intracellular enzyme nicotinamide phosphoribosyltransferase (NAMPT). NAMPT is an adipokine known to be the rate-limiting enzyme in the NAD^+^ salvage pathway and directly increases NAD^+^ levels and NAD^+^-dependent enzyme activity.^43,44^ Mechanistically, P7C3 increases NAD flux in mammalian cells and can restore cell function under conditions of overwhelming energy crisis that would normally lead to cell death.^43,45^ Regulation of the NAD^+^ cascade as a therapeutic in human disease is an area of growing interest.

Here, we describe the role of P7C3 in the prevention of IR-induced bone damage in vitro and in vivo. Most notably, P7C3 inhibited osteoclastic activity and adipogenesis and mitigated tissue loss such that bone maintained its area, architecture, and mechanical strength. The mechanistic novelty revealed is based on P7C3 significantly increasing myeloid cell differentiation and the proteins low-density lipoprotein receptor-related protein 4 (LRP-4) and transgelin (TAGLN), with downregulation of growth differentiation factor-3 (GDF-3), SH2B adapter protein 1 (SH2B1), and OX-2 membrane glycoprotein, also named cluster of differentiation 200 (CD200). Together, this pattern of protein expression may favor osteoblastic at the expense of adipogenic bone mesenchymal stem cells (BMSC) differentiation.^46–50^ TAGLN together with the upregulation of integrin-linked kinase (ILK) suggests the increased activation of actin filaments, alterations in cytoskeletal components, and anchorage-dependent cell growth, potentially enhancing cell migration and adhesion, which are important processes that control changes in cell shape, migration, proliferation, survival, and differentiation.^51^ Furthermore, the immune regulator Toll interacting protein (Tollip) and thioredoxin-binding protein-2 (TBP-2) were significantly upregulated, suggesting that excessive inflammatory resolution^52^ and reduced osteoclastogenesis via reduced RANKL^53^ activities may have also contributed to the osteoprotective effect. Finally, an important consideration is whether P7C3 also confers protection to cancer cells during IR. Here, we show that at the same protective P7C3 dose, the metabolic activity of triple-negative breast cancer and osteosarcoma cancer cell lines was significantly reduced in vitro.

## Results

### P7C3 reduces multinucleated giant cell formation and osteoclastic activity of RAW264.7 macrophages following IR in vitro

**Fig. 1:**
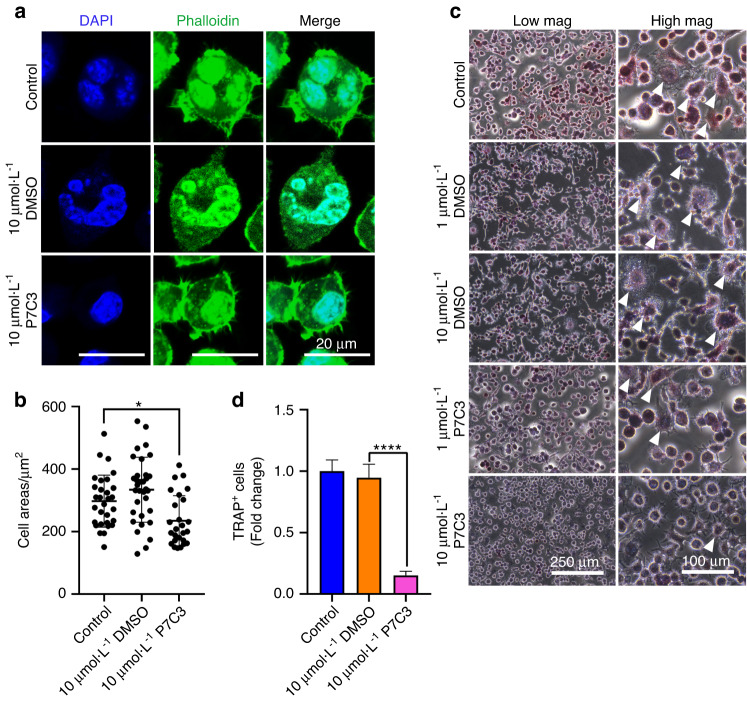
Irradiation of RAW264.7 macrophages induces the formation of multinucleated giant cells (MNGCs) 1 day post-IR (7 Gy). P7C3 treatment reduces MNGC formation and IR-associated osteoclastic activity, as indicated by reduced TRAP^**+**^ staining. Macrophage morphology was assessed via phalloidin (green, actin filaments) and DAPI (blue, cell nuclei) staining. **a** Representative confocal micrographs of RAW264.7 cells after X-ray exposure 1 day post-IR and following treatment with 10 μmol·L^−1^ DMSO or P7C3. **b** Quantification of the cell area (μm^2^) in each group. Treatment with 10 μmol·L^−1^ P7C3 reduces cell size compared with that of the control cells and following IR-induced damage. All values are given as the mean ± SE. *P < 0.05. **c** Irradiation induces the formation of “radiation-associated macrophages” (white arrows), which are characterized by the formation of multinucleated TRAP^+^ cells, suggesting the formation of cells with osteoclastic activity. On Day 3, and following supplementation of cells with 10 μmol·L^−1^ P7C3, cells displayed less intense TRAP staining compared with that of the IR-exposed group. **d** Quantification of TRAP^+^ cells. ****P < 0.000 1

We first investigated the effect of P7C3 in reducing the formation of multinucleated giant cells (MNGCs) and the osteoclastic differentiation of macrophages. Radiation-induced MNGC formation typically indicates the presence of chronic inflammation, a condition known to promote osteoclastogenesis and increase osteoclastic activity, leading to elevated bone resorption.^54^ Our previous studies demonstrated that exposure to 7 Gy IR significantly increased MNGC formation and activated osteoclastic activity in RAW264.7 macrophages.^32,55^ In this study, we assessed the ability of P7C3 to inhibit the formation of MNGCs and levels of tartrate-resistant acid phosphate^+^ (TRAP^+^) staining, which indicates activated osteoclastic activity in macrophages and following IR. Macrophages were cultured in the absence of osteoclastic differentiation factors, and our results revealed that a dose of 10 μmol·L^−1^ P7C3 significantly decreased cell size and resulted in fewer multinucleated cells than those of the IR and DMSO control groups (both *P* < 0.05; Fig. 1a, b). Furthermore, our data demonstrated fewer TRAP^+^ osteoclast-activated cells in the 10 μmol·L^−1^ P7C3 group than in all other groups (Fig. 1c). A significant reduction in TRAP^+^ cells was noted in the 10 μmol·L^−1^ P7C3-treated group compared to the control group (Fig. 1d). Our results also confirmed no change in cell morphology or size when macrophages were treated with 10 μmol·L^−1^ P7C3 in the absence of IR (Fig. S1).

### P7C3 is nontoxic and increases hBMSC and macrophage metabolic activity in vitro

**Fig. 2:**
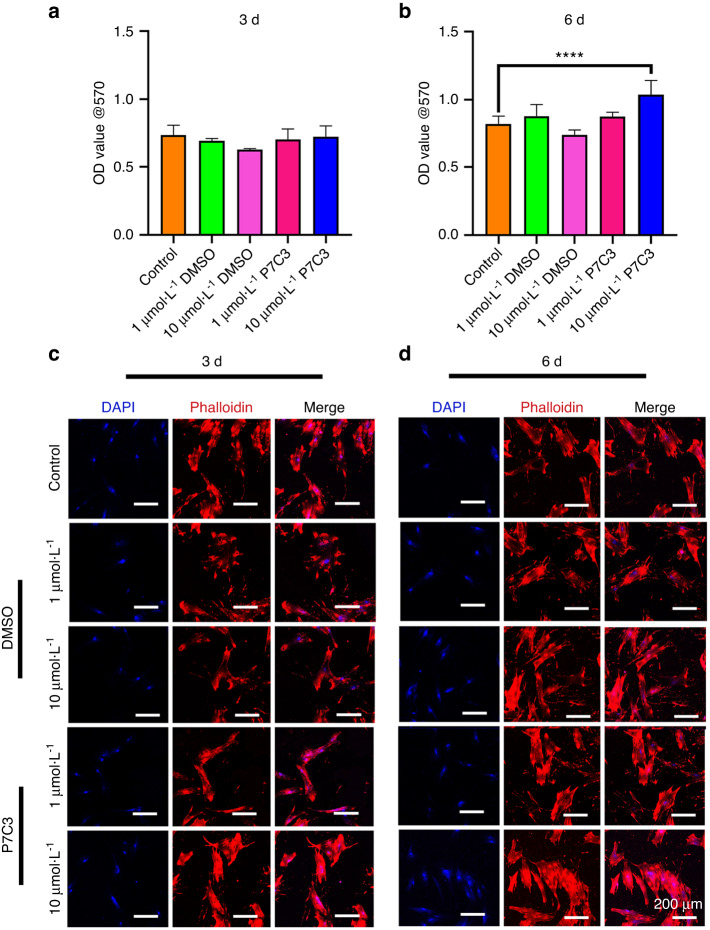
The effect of P7C3 on hBMSC metabolic activity and cellular morphology after IR. **a**, **b** Cell viability was determined by MTT assays. hBMSCs were pretreated with either 0, 1 μmol·L^−1^ or 10 μmol·L^−1^ P7C3 for 24 h before IR. After IR, cells were replenished with 1 μmol·L^−1^ or 10 μmol·L^−1^ P7C3. DMSO (1 μmol·L^−1^ or 10 μmol·L^−1^) was used as the solvent control group: ****P < 0.000 1. **c**, **d** Representative confocal micrographs of hBMSCs with or without P7C3 treatment. Cells were stained with phalloidin (red) and DAPI (blue) and were examined using confocal laser scanning microscopy

Next, we investigated the effect of P7C3 on the metabolic activity of hBMSCs and macrophages in the presence and absence of IR. The MTT assay is used to measure cellular metabolic activity as an indicator of cell viability, proliferation and cytotoxicity. When hBMSC metabolic activity was qualitatively assessed in the absence of IR, our results showed that P7C3 at both concentrations (1 μmol·L^−1^ and 10 μmol·L^−1^) was nontoxic to cells, with no apparent change in the number of live/dead cells when compared with that of the untreated control cells after 3 days of culture (Fig. S2). The effect of P7C3 on hBMSC metabolic activity after IR is shown in Fig. 2a, b. The mean OD values in the 1 μmol·L^−1^ DMSO, 10 μmol·L^−1^ DMSO, 1 μmol·L^−1^ P7C3, and 10 μmol·L^−1^ P7C3 groups and after 3 days were 0.69 ± 0.02, 0.63 ± 0.01, 0.7 ± 0.07, and 0.72 ± 0.08, respectively. No significant differences were found when these groups were compared with the IR only control cell group (0.74 ± 0.07) at this time point. However, following 6 days of culture, 10 μmol·L^−1^ P7C3 treatment resulted in a significant increase in cell growth (1.04 ± 0.1) compared to that of the IR control cells (0.82 ± 0.06, *P* < 0.000 1). hBMSC morphology was qualitatively evaluated, and as shown in Fig. 2c, d, phalloidin and DAPI staining demonstrated reduced actin filament staining on Day 3 following IR exposure. However, on Days 3 and 6, and following 10 μmol·L^−1^ of P7C3 treatment, the level and intensity of actin filaments appeared to increase compared to those of the IR control group. The effect of P7C3 on the metabolic activity of RAW264.7 cells showed a similar pattern. A significant increase in macrophage activity was found following supplementation with 10 μmol·L^−1^ P7C3 (*P* < 0.001) compared with that of the control cells on Day 3 of culture (Figs. S3A, B).

### P7C3 conferred no radioprotection to IR-induced hBMSC DNA damage in vitro

To investigate the protective effect of P7C3 on IR-induced DNA damage to hBMSCs, we measured DNA fragmentation using the alkaline Comet Assay^®^. Our previous unpublished studies have identified that maximal radiation-induced DNA damage occurred 3 days following exposure (Fig. S4). As shown in Fig. 5A–D and on Day 3 post-IR, no significant differences in the mean tail length, tail moment, and tail DNA were found when the IR, IR + DMSO, and IR + P7C3 groups were compared. On Day 6 post-IR, the mean tail length, tail moment, and tail DNA were reduced in all groups (Fig. S5E–H). These results indicate that at a concentration of 10 μmol·L^−1^, P7C3 offers limited protection against IR-induced DNA damage.

### P7C3 promotes hBMSC osteogenesis and inhibits adipogenesis following IR-induced functional damage in vitro

**Fig. 3:**
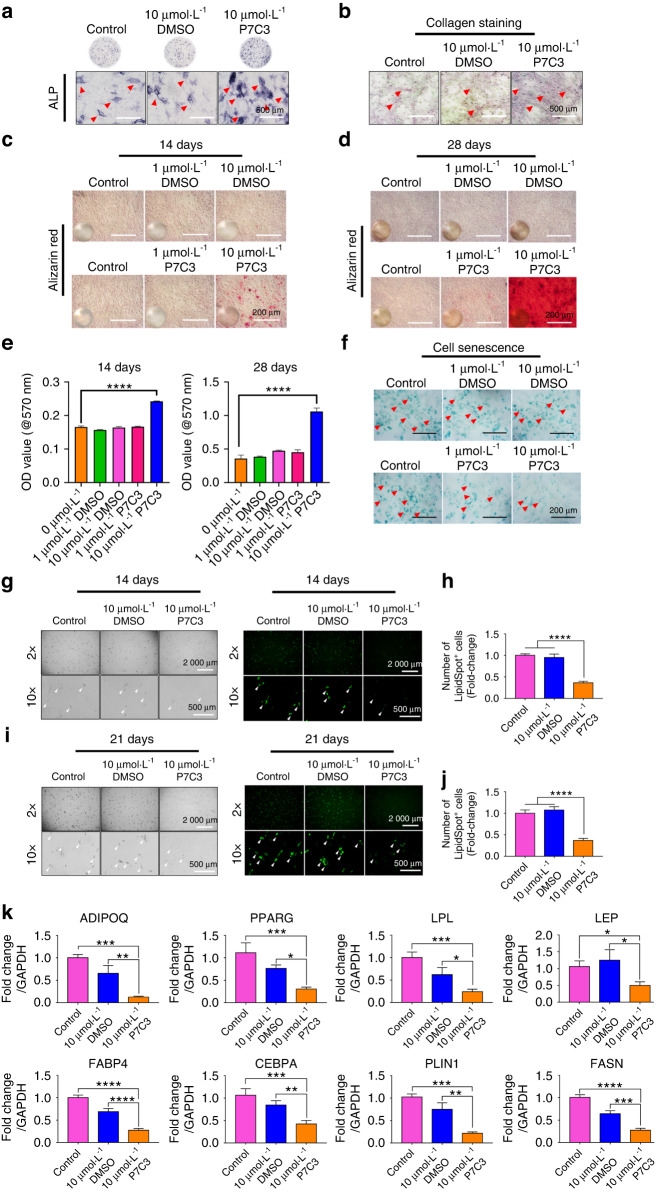
P7C3 promotes hBMSC osteogenesis and inhibits adipogenesis following IR-induced functional damage in vitro. **a** ALP-stained micrographs depicting IR-damaged hBMSCs cultured in osteogenic differentiation medium with DMSO or P7C3. **b** Representative micrographs displaying collagen formation levels. **c**, **d** Representative micrographs of Alizarin red S staining for mineralization. Mineral deposition appeared bright red in color. **e** Quantification of Alizarin red S staining: ****P < 0.000 1. **f** Representative micrographs showing senescent cells in IR-damaged hBMSCs with or without P7C3 treatment. **g** Representative phase contrast (left panel) and LipidSpot™ Lipid Droplet staining images (right panel) of hBMSCs cultured in adipogenic induction medium with 10 μmol·L^−1^ DMSO (solvent control) or 10 μmol·L^−1^ P7C3 at 14 days. **h** Quantification of LipidSpot™-positive cells at 14 days. ****P < 0.000 1. **i** Representative phase contrast (left panel) and LipidSpot™ Lipid Droplet staining images (right panel) at 21 days. **j** Quantification of LipidSpot™-positive cells at 21 days. ****P < 0.000 1. **k** qRT‒PCR showing the expression of adipogenic marker genes at 12 days post-irradiation. *P < 0.05, **P < 0.001***P < 0.001, ****P < 0.000 1

To investigate the protective effect of P7C3 on IR-induced functional damage, we assessed the osteogenic potential of hBMSCs by measuring ALP expression, neocollagenesis, and mineral deposition. On Day 14 post-IR, the levels of ALP were determined, and the results showed that the 10 μmol·L^−1^ P7C3-treated hBMSC group displayed more ALP staining than the IR only and 10 μmol·L^−1^ DMSO-treated groups (Fig. 3a). Additionally, on Day 14, collagen levels were evaluated via picrosirius red staining. As shown in Fig. 3b, increased collagen staining was observed following 10 μmol·L^−1^ P7C3 treatment compared with that of the IR-only and solvent-treated groups.

On Days 14 and 28, Alizarin red S staining was used to detect mineral deposition. As presented in Fig. 3c, d, the 10 μmol·L^−1^ P7C3-treated cells produced a larger area of mineralized nodule formation (red) than both the IR- and DMSO-treated cells. Quantitative analysis revealed that 10 μmol·L^−1^ P7C3-treated hBMSCs resulted in a significant >1.5-fold (0.24 ± 0.005, *P* < 0.000 1) increase in new bone mineral at 14 days and a > 3-fold (1.06 ± 0.05, *P* < 0.000 1) increase at 28 days post-IR compared with those of the control group (0.36 ± 0.05) (Fig. 3e), despite a harmful IR insult. Representative phase contrast images of hBMSCs with mineralized nodule formation following P7C3 treatment are shown in Fig. S6.

The persistence of unrepaired DNA damage leads to senescent cell cycle arrest.^56^ Senescent cells cause damage to surrounding healthy cells via a bystander effect and through the release of various mediators known as the senescence-associated secretory phenotype (SASP). We next assessed whether P7C3 treatment protected cells against IR-induced cell senescence using SA-β-Gal staining. As shown in Fig. 3f, the cells exposed to IR and 10 μmol·L^−1^ P7C3 treatment showed markedly decreased β-gal-positive staining compared with the IR only or DMSO-treated cells. Additionally, 1 μmol·L^−1^ P7C3-treated hBMSCs reduced β-gal-positive staining following exposure to IR, indicating decreased IR-induced cell senescence.

The adipogenic potential of hBMSCs was assessed by measuring lipid droplet formation and adipogenic-related marker gene expression. On Days 14 and 21 post-IR and in the irradiated cell group that received no P7C3 treatment, the formation of lipid droplets was pronounced, while the 10 μmol·L^−1^ P7C3-treated hBMSC group displayed significantly decreased (*P* < 0.001) staining (Fig. 3g, h). Additionally, lipid droplet staining on Day 21 showed the same trend (Fig. 3i, j). The expression levels of the adipogenesis-related marker genes *adiponectin* (*ADPOQ), PPARG, lipoprotein lipase (LPL), leptin (LEP), fatty acid binding protein 4 (FABP4), CCAAT/enhancer-binding protein alpha (CEBPA), perilipin-1 (PLIN1)*, and *fatty acid synthase (FASN)* were investigated on Day 12 following IR exposure. qRT‒PCR results demonstrated a significant reduction following P7C3 treatment in LEP (*P* < 0.05), ADPOQ, PPARG, LPL, CEBPA, PLIN1 (all *P* < 0.001), FABP4 and FASN (both *P* < 0.000 1) expression compared with that of the control group (Fig. 3k). Comparison between 10 μmol·L^−1^ DMSO and 10 μmol·L^−1^ P7C3 also showed a similar trend. In support of this result, representative phase contrast images of hBMSCs on Day 12 showing reduced lipid droplet formation following P7C3 treatment are shown in Fig. S7.

### Pretreatment and daily P7C3 reduces IR-induced bone loss and maintains bone area in vivo

**Fig. 4:**
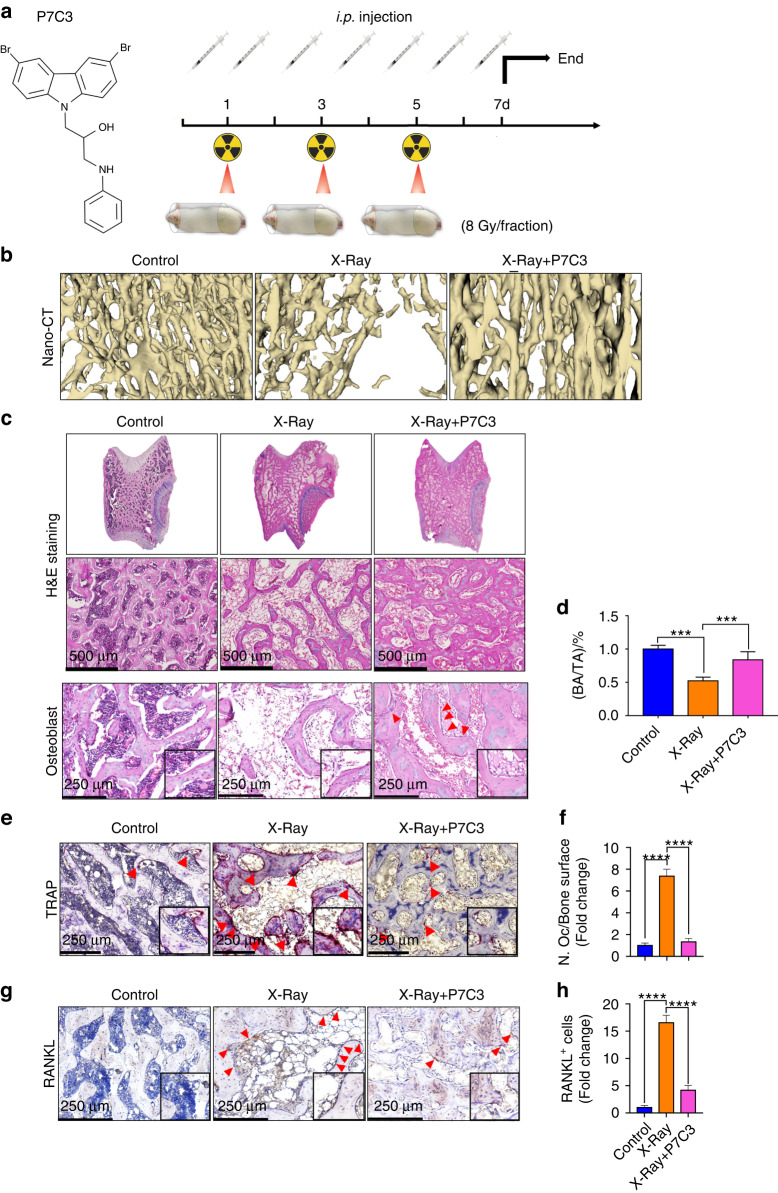
Analysis of the radioprotective effect of *i.p.* given P7C3 (20 mg·kg^−1^) following exposure to irradiation in vivo. **a** A flow chart of the animal experiment. **b** Three-dimensional models of the proximal tibia were generated using 3D Slicer™ (v4.11.20210226; Brigham and Women’s Hospital and Massachusetts Institute of Technology). The IR-treated control group showing osteoporosis. Bone architecture and area were maintained in the P7C3 + IR-treated rats. **c** Representative images of H&E-stained transverse sections through the femoral condyle and osteoblast cells (red arrow). Exposure of rats to harmful levels of irradiation resulted in bone loss and osteoporosis, as indicated by reduced trabecular connectivity, thinner and shorter trabeculae, and increased bone marrow adiposity. P7C3 treatment (20 mg·kg^−1^) markedly protected against bone loss. **d** BA/TA% quantification. ***P < 0.001. **e** Multinucleated osteoclasts were identified by TRAP staining. Red arrows indicate TRAP^+^ cells (purple‒red color). **f** The number of osteoclasts *per* unit of bone surface (cells per mm^2^) was quantified via bone histomorphometric analyses (5 images/rat, *n* = 6). ****P < 0.000 1. **g** Representative images of RANKL immunohistochemical staining. Red arrows indicate RANKL^+^ cells. **h** Quantification of RANKL^+^ cells. RANKL was strongly expressed in irradiation-exposed rats, while 20 mg·kg^−1^ P7C3 treatment reduced RANKL expression. ****P < 0.000 1

Having established the favorable impact of P7C3 on cells in vitro, we next analyzed its impact in vivo. We utilized a rat hind limb model and applied a hind limb localized accumulative IR dose of 24 Gy (three treatments of 8 Gy/dose) (Fig. 4a). For comparison, the clinical IR dose given to cancer patients varies according to cancer type and patient-specific factors (e.g., ~40–60 Gy in breast cancer patients^57^ and 50–70 Gy in head and neck patients^58,59^). In rats, 7 Gy/day for 5 days (35 Gy) is the human equivalent to a 70 Gy dose.^60^ Therefore, the exposure of rats to 8 Gy on Days 1, 3 and 5 represents a hypofractionated model delivering a human equivalent of 48 Gy (total). This, for example, represents the hypofractionated dose delivered to lung^13^ or breast cancer patients,^57^ where nontraumatic rib fracture can commonly occur following IR,^61,62^ and hypofractionated IR increases this risk.^63^ Although the risk of late-onset fragility fracture is heightened in patients,^64,65^ in rodent models, and similar to other published studies,^66,67^ our previous research showed significant levels of cell dysfunction and bone damage as early as 7 days following IR^32^.

To validate IR-induced bone loss and whether P7C3-treated rats were able to maintain their bone structure, we qualitatively examined histomorphological bone parameters using nano-CT analysis. As shown in Fig. 4b, a total of 24 Gy of radiation resulted in significant bone tissue loss as expected,^32^ while 20 mg·kg^−1^ P7C3-treated rats showed reduced bone loss compared to the IR-treated rats. Skeletal changes were further assessed histologically and by H&E staining. As shown in Fig. 4c, rats developed osteoporotic-like bone features characterized by substantial deterioration of bony architecture, reduced trabecular connectivity, thinner and shorter trabeculae, and increased bone marrow adiposity. In contrast, the 20 mg·kg^−1^ P7C3-treated and IR-exposed rats exhibited marked improvement, with results demonstrating a bony structure similar to that of the non-IR exposed, healthy control group. Additionally, qualitative histological analysis showed more and larger, darkly stained cuboidal osteoblasts on the bone surface following 20 mg·kg^−1^ P7C3 treatment. This finding suggests the presence of activated osteoblasts and was noted to be in contrast to the quiescent bone surfaces observed in the control and IR-treated groups. Quantification of %bone area/total area (BA/TA%) (Fig. 4d) demonstrated a significant reduction in bone area in the IR-exposed animals at 7 days compared to IR-exposed P7C3-treated animals (*P* < 0.001).

### P7C3 reduces osteoclastic activity and cell staining for the osteoclastogenesis factor RANKL^+^ following exposure to harmful levels of IR in vivo

To evaluate whether P7C3 treatment regulates osteoclast activation and a key osteoclastogenesis factor, RANKL, we prepared transverse sections through the femoral condyle and performed TRAP staining. Staining revealed that the number of TRAP^+^ and therefore active osteoclasts was significantly higher in the IR-induced rats than in the control animals (*P* < 0.000 1). Furthermore, the number of active osteoclasts was significantly reduced following P7C3 treatment compared with that of the IR-exposed control group (*P* < 0.000 1; Fig. 4e, f). Similarly, immunohistochemical analysis also revealed a significant >3.5-fold decrease in the number of RANKL^+^ cells in the P7C3-treated animals compared to the control animals (*P* < 0.000 1; Fig. 4g, h). Altogether, these results suggest that 20 mg·kg^−1^ P7C3 treatment may decrease the activation of osteoclastic resorption and regulate osteoclastogenesis by reducing the number of RANKL^+^ cells despite exposure to harmful levels of irradiation.

### P7C3 maintained bone strength (ultimate stress (σu) and fracture stress (σf)) despite exposure to a harmful IR insult in vivo

**Fig. 5:**
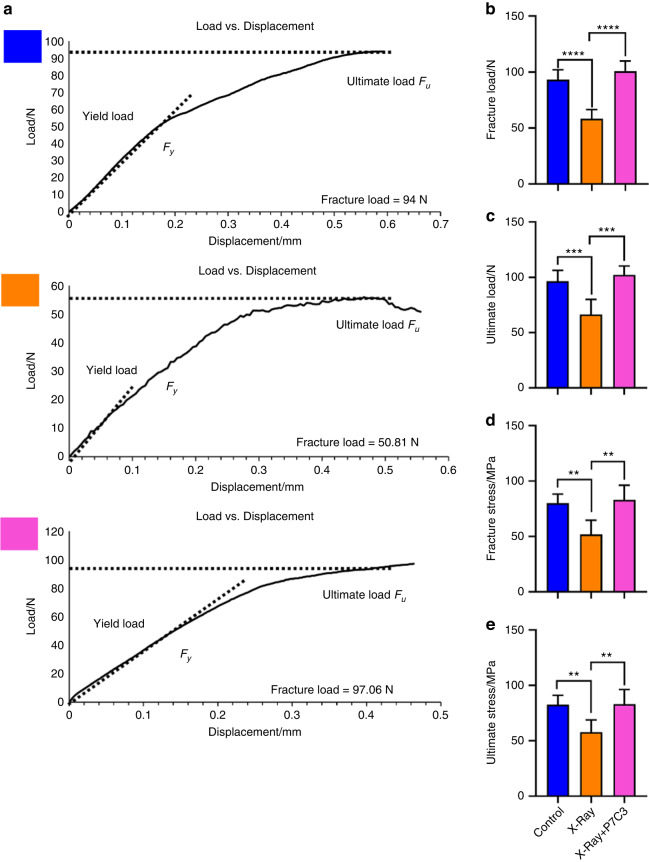
P7C3 maintains the strength of bone despite exposure to harmful levels of irradiation (*n* = 6). **a** Representative load‒displacement curves of the nonirradiated healthy control, IR-exposed, and IR + P7C3 groups. The mechanical properties of fracture load (**b**), ultimate load (**c**), fracture stress (**d**), and ultimate stress (**e**) at the tibial midpoint varied between the different groups. Animals receiving 20 mg·kg^−1^ *i.p*. treatment showed higher fracture load/stress and ultimate load/stress, suggesting that P7C3 protects bone strength despite exposure to harmful irradiation. **P < 0.01, ***P < 0.001, ****P < 0.000 1

Next, we evaluated whether the P7C3-treated rats were able to maintain their cortical bone structure and strength. The 3-point bending test fixture is presented in Fig. S8. Representative stress‒displacement deformation plots for tibiae in the control, IR, and IR + P7C3 groups are shown in Fig. 5a. The tibiae exhibited varying deformation behavior when tested under 3-point bending. Rats subjected to IR resulted in a significant decrease in fracture load (*P* < 0.000 1; Fig. 5b), ultimate load (*P* < 0.001; Fig. 5c), fracture stress (*P* < 0.01; Fig. 5d), and ultimate stress (*P* < 0.01; Fig. 5e), indicating increased fragility and failure under low loads compared to those of the healthy control animal group. However, the animals treated with P7C3 maintained bone strength to similar levels to the non-IR healthy control animals. Tibiae in the IR + P7C3 group displayed a significantly increased fracture load (*P* < 0.000 1, 95.37 ± 2.24 N), ultimate load (*P* < 0.001, 97.21 ± 1.54 N), fracture stress (*P* < 0.01, 77.94 ± 17.28 MPa), and ultimate stress (*P* < 0.01, 78.64 ± 16.08 MPa) compared to those of the IR only group (58.20 ± 8.33 N (fracture load); 66.25 ± 13.77 N (ultimate load); 51.65 ± 13.06 MPa (fracture stress), and 57.71 ± 11.15 MPa (ultimate stress)). Additionally, no significant differences were found between the non-IR control animals and the IR + P7C3 animals. These results show that IR negatively impacts bone strength, making it more susceptible to fracture. Remarkably, treatment with P7C3 had a significant and positive impact by maintaining a healthy level of bone strength despite exposure to harmful levels of IR.

### P7C3 induces alterations in serum cytokine/chemokine protein profiles in vivo

**Fig. 6:**
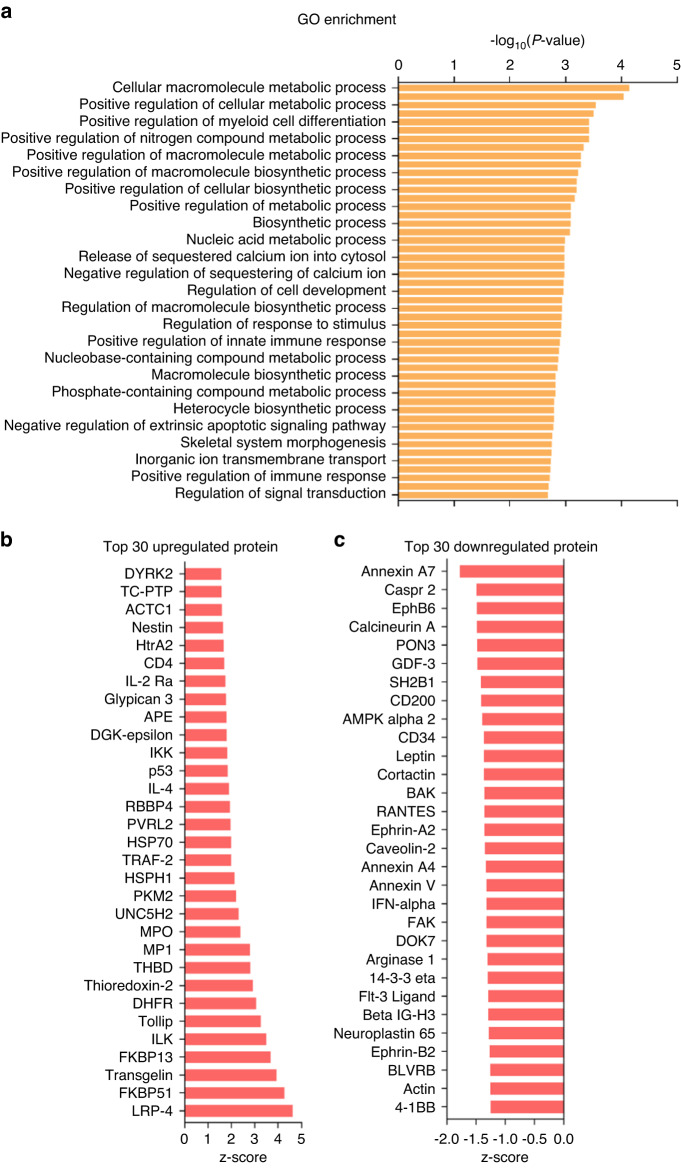
Serum cytokine and chemokine profiles following P7C3 treatment and IR. **a** GO enrichment analysis. The top 30 most significantly enriched GO terms following P7C3 treatment are presented. **b** The top 30 differentially expressed proteins (upregulated) following P7C3 treatment are presented. **c** The top 30 differentially expressed proteins (downregulated) following P7C3 treatment are presented

To better understand the systemic effect of P7C3 in irradiated animals, we quantified cytokine and chemokine expression in serum using a cytokine array that examined 500 rat proteins simultaneously. Hierarchical clustering of the 500 biomarkers is shown in Fig. S9. GO enrichment analysis was first performed to investigate the functional classification of these proteins. As shown in Fig. 6a, the top 10 highly enriched GO terms were cellular macromolecule metabolic process, positive regulation of biosynthetic process, positive regulation of cellular metabolic process, maintenance of location in cell, positive regulation of myeloid cell differentiation, positive regulation of protein catabolic process, positive regulation of nitrogen compound metabolic process, regulation of extrinsic apoptotic signaling pathway, positive regulation of macromolecule metabolic process, and cellular response to tumor necrosis factor. Additionally, GO enrichment analysis of each cluster is presented in Fig. S10. These results indicate that these GO terms may play an important role in the radioprotective effect of P7C3 against IR-induced bone loss.

KEGG pathway analysis is presented in Fig. S11. The top 30 upregulated proteins are shown in Fig. 6b. The results showed high expression levels of the proteins LRP-4, FK506-binding protein 51 (FKBP51), TAGLN, FK506-binding protein 13 (FKBP13), ILK, Tollip, dihydrofolate reductase (DHFR), TBP-2, thrombomodulin (THBD), and melanization protease 1 (MP1) following P7C3 treatment. Annexin A7, contactin-associated protein-2 (Caspr 2), ephrin type-B receptor 6 (EphB6), calcineurin A, serum paraoxonase/lactonase 3 (PON3), GDF-3, SH2B1, CD200, AMP-activated protein kinase alpha 2 (AMPK alpha 2), and sialomucin, also named cluster of differentiation 34 (CD34), were the top 10 downregulated proteins following P7C3 treatment (Fig. 6c). Additionally, the heatmaps of the top 30 highly expressed cytokines and downregulated proteins in serum following P7C3 treatment are presented in Fig. S12A, B.

### P7C3 reduces IR-induced bone marrow adiposity and lipid formation in vivo

**Fig. 7:**
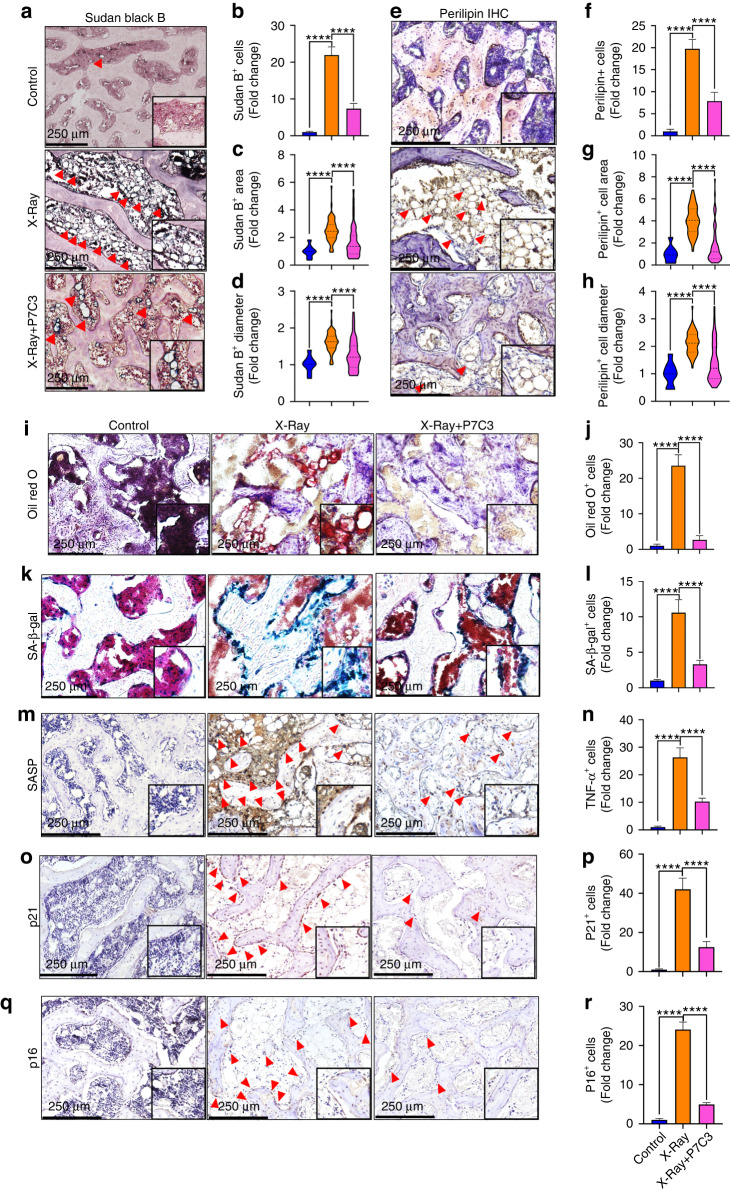
P7C3 treatment (20 mg·kg^−1^) reduces bone marrow adiposity and cell senescence following exposure to harmful irradiation (*n* = 6). **a** Representative photomicrographs of Sudan Black B staining for adipocytes and lipids (stained black). The nuclei were counterstained with nuclear fast red (pink). **b** Quantification of Sudan Black B^+^ cell number in each of the groups. **c** Quantification of Sudan Black B^+^ cellular area and **d** cell diameter. **e** Representative photomicrographs of perilipin immunohistochemical staining. Red arrows indicate perilipin^+^ cells. **f** Quantification of perilipin^+^ cells. **g** Perilipin^+^ cellular area. **h** Perilipin^+^ cell diameter. **i** Representative micrographs of Oil Red O staining of the rat bone sections. P7C3 reduced IR-induced bone marrow adiposity. **j** Quantification of Oil Red O staining. **k** Representative photomicrographs of SA-β-Gal staining for senescent cells (stained blue). **l** Quantitative analysis of SA-β-Gal^+^ cell intensity. **m** Representative images of senescence-associated secretory phenotype (SASP) TNF-α^+^ cell staining. Red arrows indicate TNF-α^+^ cells. **n** Quantification of TNF-α^+^ cells in each of the groups. ****P < 0.000 1. **o** Representative images of p21^+^ cell staining. **p** Quantification of p21^+^ cells in each of the groups. ****P < 0.000 1. **q** Representative images of p16^+^ cell staining. **r** Quantification of p16^+^ cells in each of the groups. ****P < 0.000 1

Our in vitro data showed that P7C3 inhibited adipogenesis, and the cytokine array results revealed that P7C3 upregulated LRP-4, TAGLN, ILK, and Tollip and downregulated GDF-3, SH2B1, and CD200, uncovering many factors able to regulate preferential myeloid progenitor BMSC differentiation toward osteoblastogenesis at the expense of adipogenesis. Notably, some of these factors facilitate inflammatory resolution (Tollip) and suppress RANKL expression and osteoclastogenesis (TBP-2), thereby supporting our in vivo RANKL and TRAP staining results presented in Fig. 4. Therefore, bone marrow adipogenesis and the presence of lipids such as phospholipids, sterols and neutral trigycerides were further investigated using Sudan black B staining. As shown in Fig. 7a, bone marrow adipocytes were nearly absent in the non-IR control group. In contrast, the IR-exposed rats displayed an intense number of positively stained adipocytes and lipids. The P7C3-treated group showed less Sudan black B^+^ staining. Quantification of positively stained adipocytes demonstrated a significant and >20-fold increase in adipocyte number in the IR group compared with the control group (*P* < 0.000 1, Fig. 7b). Although the P7C3-treated animals displayed an ~5-fold increase in adipocyte number compared to the controls, this value was significantly less (~15-fold) than that of the untreated, IR-exposed animals (*P* < 0.000 1). The results also revealed an IR-induced increase in adipocyte area (*P* < 0.000 1, Fig. 7c) and diameter (*P* < 0.000 1, Fig. 7d) compared to those of the control animals. However, treatment with P7C3 significantly reduced both adipocyte area (*P* < 0.000 1) and diameter (*P* < 0.000 1) compared with those of the IR-exposed animals. Together, these results indicate a reduction in lipid accumulation and bone marrow adiposity (as defined by a reduction in the proportion of the bone marrow cavity volume occupied by adipocytes) following P7C3 treatment.

Changes in adiposity and lipid droplet formation were further investigated via perilipin immunohistochemistry and Oil Red O staining, respectively. The results revealed a significant IR-induced increase in perilipin^+^ cells compared with those of the non-IR control animals (~19-fold; *P* < 0.000 1). However, P7C3 treatment resulted in a significant reduction in perilipin^+^ cell number (*P* < 0.000 1, Fig. 7e, f), cell area (*P* < 0.000 1, Fig. 7g), and cell diameter (*P* < 0.000 1, Fig. 7h) compared to those of the IR-exposed animals. Similarly, quantification of frozen Oil Red O-stained sections revealed a significant >21-fold increase in lipid formation in the IR-exposed animals compared to the control animals (*P* < 0.000 1). Importantly, P7C3 treatment significantly reduced lipid staining ~20-fold (*P* < 0.000 1) compared to that in the animals in the IR group (Fig. 7i, j).

### P7C3 reduces IR-induced cellular senescence in vivo

To further determine the protective effect of P7C3 against IR-induced cellular senescence, we analyzed SA-β-gal activity. Our findings show a significant ~10-fold increase in senescence within bone marrow cells in response to IR (*P* < 0.000 1); however, analyses showed significantly reduced SA-β-gal^+^ activity in the P7C3-treated rats compared to the IR-treated rats (*P* < 0.000 1, Fig. 7k, l). Similarly, quantitative immunohistochemical analysis showed that IR induced a significant increase in SASP TNF-α^+^, p21^+^, and p16^+^ cells compared to those of the control animals (*P* < 0.000 1). A significant reduction in SASP TNF-α^+^, p21^+^, and p16^+^ cells were found in the P7C3-treated animals compared to the control IR-treated animals (*P* < 0.000 1; Fig. 7m–r).

### P7C3 inhibits MDA-MB-231, PC3, and OS cancer cell metabolic activity in a dose-dependent manner in vitro

Our results have shown that P7C3 appears nontoxic to host cells and radioprotects against IR-induced cell damage, tissue loss, and bone fragility. However, it is critical to determine whether a protective effect is also afforded to cancer cells. To this end, we quantified changes in the metabolic activity of MDA-MB-231, PC3, and OS cells in response to increasing concentrations (10 μmol·L^−1^, 20 μmol·L^−1^, and 40 μmol·L^−1^) of P7C3 ± IR in vitro.

### Triple-negative breast cancer (MDA-MB-231-GFP) cell metabolic activity ± IR in vitro

**Fig. 8:**
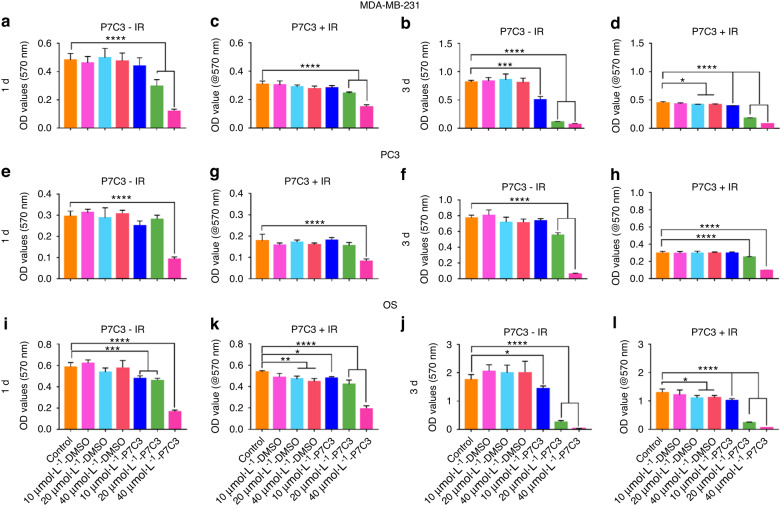
The effect of P7C3 on the metabolic activity of MDA-MB-231, PC3, and hOs cancer cell lines ± IR. **a**, **b** Cell activity of MDA-MB-231-GFP cells following P7C3 treatment. MDA-MB-231-GFP cells were exposed to increasing concentrations of P7C3 or solvent control. ***P < 0.001, ****P < 0.000 1. **c**, **d** Cell activity of MDA-MB-231-GFP cells following P7C3 + IR treatment. MDA-MB-231-GFP cells were exposed to 7 Gy IR followed by incubation with increasing concentrations of P7C3 or solvent control. *P < 0.05, ****P < 0.000 1. **e**, **f** Cell proliferation of PC3 cells following P7C3 treatment. ****P < 0.000 1. **g**, **h** Cell proliferation of PC3 cells following P7C3 + IR treatment. PC3 cells were exposed to 7 Gy IR followed by incubation with increasing concentrations of P7C3 or solvent control. ****P < 0.000 1. **i**, **j** Cell activity of OS cells following P7C3 treatment. The OS cells were exposed to increasing concentrations of P7C3 or solvent control. *P < 0.05, ***P < 0.001, ****P < 0.000 1. **k**, **l** Cell activity of OS cells following P7C3 + IR treatment. The OS cells were exposed to 7 Gy IR followed by incubation with increasing concentrations of P7C3 or solvent control. *P < 0.05, **P < 0.01, ****P < 0.000 1

As shown in Fig. 8a–d, exposure of MDA-MB-231 cells to IR resulted in a significant decrease in cell activity. The mean OD value measured in the control group on Day 1 (-IR) was 0.48 ± 0.05, which was reduced to 0.32 ± 0.03 following exposure to IR (*P* < 0.01), a reduction in growth of 34.7%. On Day 3, cell metabolic activity was reduced by 43.8% when IR was applied. Remarkably, our results revealed that in the absence of IR and on Day 1 of culture, P7C3 at concentrations of 20 μmol·L^−1^ and 40 μmol·L^−1^ significantly reduced MDA-MB-231-GFP cell activity by 40.0% and 80.0%, respectively (*P* < 0.000 1 in both groups). By Day 3 of culture, cell activity was also significantly reduced (37.5%) in the 10 μmol·L^−1^ P7C3 group (*P* < 0.001) compared with the control cell group. At this time point, cell activity was further reduced in the 20 μmol·L^−1^ and 40 μmol·L^−1^ groups by 86.3% and 91.3%, respectively. The inhibitory activity of P7C3 on MDA-MB-231-GFP cells was maintained following exposure to IR; however, no radiosensitizing effect was measured. Live cell imaging revealed a marked decrease in MDA-MB-231-GFP cell activity following all doses of P7C3 ± IR, especially at the higher concentrations of 20 μmol·L^−1^ and 40 μmol·L^−1^ (Fig. S13A–D). The results showed that MDA-MB-231-GFP cells were sensitive to IR and displayed a dose-dependent inhibitory response to P7C3. Furthermore, MDA-MB-231-GFP spheroids were cultured, and the spheroid area (μm^2^) was measured and compared between groups (Fig. S14). The results showed a significant and >2-fold reduction in spheroid size following P7C3 treatment at each of the three doses compared with that of the nontreated control group (*P* < 0.000 1 in all groups).

### Metastatic prostate cancer (PC3) cell metabolic activity ± IR in vitro

As shown in Fig. 8e–h, the exposure of PC3 cells to IR in the absence of P7C3 resulted in a significant and negative impact on cell activity. On Day 1, a mean OD of 0.30 ± 0.02 (−IR) was reduced to 0.18 ± 0.03 (+IR; *P* < 0.01), signifying an IR-induced decrease in activity of 40%. On Day 3, and following IR exposure, activity had further decreased to 57.1% (*P* < 0.000 1) compared with that of the control non-IR cells. Following supplementation with P7C3 in the absence of IR, a trend of decreased PC3 activity was observed with a significant reduction measured in the 40 μmol·L^−1^ group (*P* < 0.000 1) on Day 1 and in the 20 μmol·L^−1^ and 40 μmol·L^−1^ groups on Day 3 (both *P* < 0.000 1). Similar to that of the MDA-MB-231-GFP group, the inhibitory activity of P7C3 was maintained in the presence of IR; however, no synergistic effect was measured. Live cell imaging revealed a dose-dependent inhibitory response to P7C3 ± IR (Fig. S15A–D).

### Osteosarcoma (OS) cell metabolic activity ± IR in vitro

Next, we investigated the effect of P7C3 on OS cell growth (Fig. 8i–l). When control cell activity was compared (−IR vs. + IR), the results demonstrated a significant IR-induced 27.0% decrease on Day 3 (*P* < 0.001). Furthermore, our results showed that when OS cells were treated with P7C3 at concentrations of 10 μmol·L^−1^, 20 μmol·L^−1^, and 40 μmol·L^−1^ in the absence of IR, a significant decrease in cell activity was found on Day 1 (*P* < 0.000 1 in all groups) and Day 3 (10 μmol·L^−1^; *P* < 0.05, and 20 μmol·L^−1^ and 40 μmol·L^−1^; *P* < 0.000 1). No synergistic effect was found when P7C3 was combined with IR. Live cell imaging revealed a dose-dependent inhibitory response to P7C3 ± IR (Fig. S16A–D).

## Discussion

Ionizing radiation exposure is relevant to cancer patients undergoing radiotherapy,^5,7,12–14,20,68^ astronauts,^31,69,70^ radiation workers and victims of nuclear accidents.^71–73^ Presently, radiotherapy-induced adjuvant damage to otherwise healthy bone tissue is a major cause of pain and morbidity,^5,7,10,74^ and no effective therapeutic strategy exists. Therefore, the development of a novel osteoprotectant is urgently needed. P7C3, when administered via intraperitoneal, intravenous or oral routes, has been found to have a favorable half-life and volume of distribution and could be safely administered to rodents for prolonged periods and in relatively high concentrations.^43,75^ To the best of our knowledge, the efficacy of P7C3 toward bone tissue, in vitro, in vivo or post-IR, and its effect on MDA-MB-231, PC3, and OS cancer cell lines are unknown. Here, we identify P7C3 as a novel therapeutic radioprotective strategy for bone.

Cellular DNA is highly vulnerable to IR via direct damage and indirect ROS-mediated damage.^76^ This damage subsequently triggers cell cycle arrest and suppressed proliferative potential, leading to functional damage to the cell.^77^ Through ROS-activated NF-κB signaling, SASP is also able to induce DNA damage in healthy cells, thus further facilitating senescence.^78^ Furthermore, IR and SASP exposure to BMSCs leads to a reduction in their capacity for osteogenic commitment.^79–81^ Similar to our previous studies,^32,55^ the results here also confirmed markedly increased IR-induced BMSC DNA damage and fragmentation. Our data also confirmed that on Days 3 and 6 post-IR, pretreatment with P7C3 provided no protection against DNA damage in vitro. Intriguingly, despite the DNA damage measured, P7C3-treated cells displayed fewer IR-induced senescent cells, increased ALP, neocollagenesis, and osteogenesis, with significantly increased bone mineral deposition in vitro. Furthermore, our in vivo investigations showed that IR induced an ~25-fold increase in SASP, and this effect was significantly reduced ~15-fold in the P7C3-treated animal group. The mechanism(s) through which P7C3 was able to decrease senescence and increase osteogenic commitment despite DNA damage remains unclear. This phenomenon may be explained in part by the p53 protein. Bai and colleagues^79^ showed that IR-induced DNA damage suppressed cell viability and osteogenic differentiation potential and facilitated senescence via p53/p21 senescence-related gene expression. Our microarray results revealed the substantial upregulation of p53 in the P7C3-treated animals compared to the control IR animals. p53 is coupled to various post-translational modifications (e.g., phosphorylation and acetylation), critical steps in mediating the cellular response to IR-induced DNA damage. Furthermore, p53 activates de novo synthesis of proapoptotic molecules, and in this study, its upregulation may have reduced the formation of cellular senescence and instead favored apoptosis or cell survival where damaged DNA can be subsequently repaired.^82,83^ However, this is cautiously speculated, as the differential activation of cell cycle arrest or cell death by p53 is not completely understood, and p53 protein levels have been shown to be proportional to the extent of DNA damage in a cell.^84^ It is also important to highlight that our results reflect the short-term (7 days) post-IR, and cells that survive immediate genotoxic IR stress display genomic instability up to 36 days post IR.^85–88^ Thus, any dysfunction may not be observed until longer after IR exposure.^85,89^ As such, further longer-term studies are warranted. Finally, although supplementary data showed that P7C3 offered no protection against IR-induced DNA damage, it is important to acknowledge that further investigation at earlier 4–8-h time points post-exposure, and when most DNA damage occurs, is needed.

An increase in bone marrow adiposity has been observed in most bone loss conditions, including aging^90,91^ and various pathological conditions.^91–96^ Bone marrow adipocytes are derived from the same progenitor cells (e.g., BMSCs) as osteoblasts. A switch in lineage commitment toward adipogenic differentiation at the expense of osteogenic differentiation following IR-induced injury^65^ or via transdifferentiation of osteoblasts into adipocytes^66^ has been identified. Interestingly, IR exposure directly and preferentially reduced the osteogenic potential of BMSCs compared to more resistant adipogenic differentiation.^97,98^ However, the underlying mechanisms involved are yet to be clarified. Our in vitro studies show that supplementation of cells with P7C3 significantly reduced IR-induced master adipogenic transcription factor *PPARγ* expression, along with other key adipogenic transcription factors, and with significantly reduced levels of lipid droplet formation. Notably, a >2-fold increase in bone mineral deposition was found in the P7C3-treated cells compared to the control IR-exposed cells. Our in vivo findings further support preferential IR-induced adipocyte formation and the antiadipogenic activity exerted following P7C3 treatment. Seven days following IR exposure, an ~20-fold increase in bone marrow adiposity and lipid formation was measured in the control group. Notably, pretreatment and daily *i.p*. administration of P7C3 significantly reduced the aberrant changes in the number and size of bone marrow adipocytes and lipid formation, despite exposure to harmful levels of IR. This finding indicates the potential of P7C3 to play a critical role in the suppression of adipogenesis alongside preferentially enhancing osteoblastogenesis. Although bone was able to maintain its structure and strength, new bone formation in vivo was not measured in this study and warrants further important investigation in future studies.

Notably, the Wnt/β-catenin signaling pathway is a well-studied endogenous regulator of mesenchymal cell fate. Decreased Wnt/β-catenin signaling is a key step in the commitment of progenitor cells to the adipogenic lineage, adipocyte maturation, and de novo lipogenesis, while upregulation promotes osteoblastogenesis.^99^ Five hundred serum proteins were screened following in vivo exposure to IR ± P7C3. The results revealed that exogenous administration of P7C3 reduced the expression of proteins related to adipogenesis and upregulated those associated with promoting osteogenesis. For example, the protein LRP-4 was strongly upregulated in the P7C3-treated animals post-IR. LRP-4 is highly expressed by osteoblasts and early osteocytes and is considered to play an important role in bone metabolism and homeostasis, potentially through interaction with SOST/sclerostin.^100^ PPARγ is essential for the production of sclerostin,^101^ a protein that mediates endocrine communication between fat and bone tissue, and its expression was significantly inhibited by P7C3. Sclerostin increases adipogenesis while decreasing osteogenesis via Wnt/β-catenin signaling activation.^102,103^ Interestingly, Bullock et al.^104^ reported the prevention of disuse osteoporosis in an LRP-4 knock-in murine model. The authors concluded that LRP-4 may serve as a high yield target for the development of compounds that protect skeletal integrity. Paradoxically, Chang et al.^100^ demonstrated that LRP-4 deficiency resulted in increased sclerostin serum levels and a progressive gain in cancellous and cortical bone as measured in a LRP-4 knockout murine model. A study by Kim et al.^46^ demonstrated the differential impact of LRP-4 and sclerostin expression in adipocytes vs. osteoblasts and concluded that the impact on adipocyte physiology was distinct from the effect on osteoblast function. With the support of our findings, these studies highlight a key and multifaceted role of LRP-4 in bone regulation and an emerging area that warrants further research.

Similarly, the protein TAGLN was upregulated. Transgelin is a transforming growth factor beta-inducible gene and is considered a novel regulator of BMSC commitment to the osteogenic and adipogenic lineage. Transgelin regulates differentiation via the distribution of actin filaments and alterations in cytoskeletal organization.^105^ This is important, as during lineage commitment, BMSCs undergo a significant modification in morphology and actin cytoskeletal organization, which participates in cell fate determination.^106^ Furthermore, actin levels can directly influence osteogenesis vs. adipogenesis via Yes-associated protein and the regulation of Runx2,^107^ as well as during proliferation.^108^ Elsafadi et al.^47^ reported that TAGLN overexpression enhanced the osteogenic and adipogenic differentiation of hBMSCs, together with their migration in vitro. Increased bone formation was also reported in vivo. TAGLN deficiency resulted in impaired osteogenic and adipogenic differentiation and reduced cell motility, which was determined to be due to downregulation of the actin cytoskeletal and focal adhesion pathways. Furthermore, and in vivo, BMSCs are not in isolation but physically interact with components in the microenvironment, including mechanical stimuli that influence osteogenic vs. adipogenic differentiation.^109^ To this end, mechanical promoters (e.g., varying geometries and fluid flow) of cytoskeleton contractility are considered osteogenic, while those disrupting contractility promote adipogenesis.^110^ Our findings suggest that TAGLN plays a key role in the osteoprotective response observed, and this may occur through the optimal activation of actin filaments, alterations in cytoskeletal components, and potentially enhanced cell migration and adhesion. This finding is further supported by the strong upregulation of ILK, a protein also involved in cell-matrix interactions, cell adhesion, and anchorage-dependent cell growth.^111^ Important processes that control cell shape change, migration, proliferation, survival, and differentiation via interaction with the actin cytoskeleton.^51^

Our results also reveal substantial downregulation of GDF-3. GDF-3 is highly expressed in adipose tissue macrophages and adipocytes and has been reported to play a major role in regulating adipose tissue homeostasis and energy balance.^112^ Low levels of GDF-3 expression may have further facilitated osteogenic commitment vs. adipogenesis. For example, Shen et al. ^48^ reported that GDF-3 deficiency protected mice against diet-induced obesity. Other supporting studies have demonstrated that overexpression of GDF-3 via PPAR*γ* suppressed lipolysis, facilitated adipose accumulation,^113^ and increased adipose-tissue mass and weight gain in mice.^114^ Finally, P7C3 treatment was also associated with substantially reduced levels of SH2B1 and CD200, two proteins associated with increasing the level of tissue adiposity.^49,50^

A further consequence of IR exposure is cell dysfunction due to increased levels of inflammatory cytokines (e.g., TNFα, IL-1β, and IL-6),^115^ which play a pivotal role in the promotion of osteoclastogenesis, osteoclastic activity, and subsequent bone resorption.^116^ Our in vitro results showed that P7C3 prevented macrophage MNGC formation, a hallmark indicator of chronic inflammation. Notably, in vivo P7C3 facilitated the strong upregulation of Tollip, a multifunctional immune regulator that prevents an excessive proinflammatory response.^52^ Under strong and acute inflammatory conditions, Tollip serves as a negative inhibitor of the NF-κB signaling pathway and facilitates the resolution of inflammation, promoting autophagy and clearance of cellular debris as well as excessive lipids by enabling vacuole transport.^117,118^ Thus, Tollip may also contribute a critical osteoprotective response against IR-induced damage. Our in vivo findings also demonstrated a significant reduction in the number of RANKL^+^ cells and active TRAP^+^ osteoclasts in the P7C3-treated animals post-IR. Notably, TBP-2 was also strongly upregulated following P7C3 administration. For example, Aitken et al. ^53^ demonstrated that TBP-2 overexpression inhibited osteoclastogenesis, potentially via RANKL signaling events. Thus, TBP-2 may also play an important role in regulating bone-resorbing osteoclasts following IR insult. Notably, TBP-2 is also a key regulator of lipid and glucose metabolism; however, the mechanism remains elusive.^119^

Mechanistically, P7C3 increases intracellular NAD flux through overexpression of NAMPT. NAMPT has been shown to promote osteogenesis via increases intracellular NAD^+^ levels, the NAD^+^/NADH ratio, and SIRT1 activity.^120–122^ Notably, NAMPT underexpression resulted in a reduced level of osteogenesis.^123^ Furthermore, higher levels of NAD were reported to inhibit osteoclastogenesis via altered RANKL activity, while lower levels stimulate osteoclastogenesis.^123^ Li et al.^124^ recently reported that osteogenic committed BMSCs exhibited elevated intracellular NAD^+^ levels in vitro, while a decline in intracellular NAD^+^ levels was found in adipogenic committed BMSCs. The authors concluded that NAD^+^-mediated processes were indispensable for osteogenic commitment and in bone repair. NAD^+^, NAMPT, and SIRT1 levels were not measured in this study and therefore warrant further investigation. However, these studies support the potential and important role of P7C3-driven NAD formation in osteogenesis and in contributing to the osteoprotective response observed.

Radiotherapy is mainly based on the principle that normal tissue cells exhibit a greater DNA repair capacity than carcinoma cells following IR damage. In light of the protective activity of P7C3s against host cells, it is difficult to consider that the drug may not also provide a protective response against cancer cells. However, our preliminary findings show that P7C3 had a cytotoxic effect on MDA-MB-231, PC3, and OS cancer cell lines. Chen et al. ^125^ demonstrated that P7C3, at similar doses, without damaging normal cells, suppressed the malignant growth of glioma cells in vitro and in vivo by regulating aerobic glycolysis. Cancer cells prefer glycolysis to supply advancing energy requirements under normal oxygen conditions. Therefore, this and other studies^126–129^ suggest that glycolysis is feasible for targeted therapy, and as such, P7C3 may be an important and undiscovered agent in suppressing cancer progression.

The degree of radiotherapy-induced bone damage to patients varies due to a number of factors, including sex, age, dose per fraction, total dose, chemotherapy, any comorbidities, and the cancer itself.^7,20,130^ It is therefore difficult to simulate these confounding clinical factors using our rodent model. A further limitation is that exposure of both hind limbs to IR does not mimic cancer radiotherapy where high-dose focal radiation is applied to a local region, thereby potentially altering the subsequent mechanistic cellular response to IR.^131^ Finally, due to anatomical factors, the level of IR to tissue can vary by 23%–32%.^132^ In this respect, and as a dosimeter was not used, confirmation that reproducible and accurate IR doses were delivered to bone was not provided in this study.

## Conclusion

**Fig. 9:**
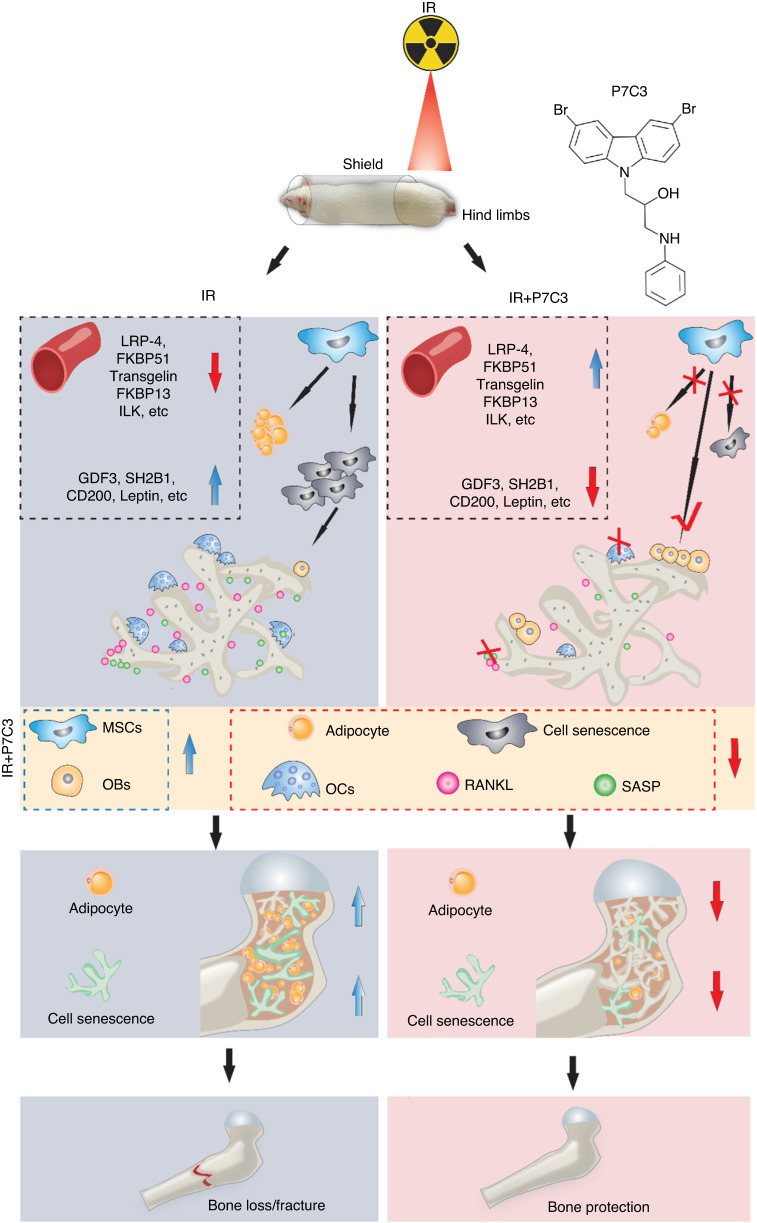
Schematic diagram highlighting the protective effect provided by P7C3 against IR-induced bone loss. Exogenous administration of 20 mg·kg^−1^ P7C3 shifts the pathological environment induced by irradiation from favoring osteoclastogenesis into osteogenesis and reduces bone marrow adipogenesis and cell senescence, thereby significantly protecting bone from IR-mediated bone loss and fracture in vivo

Controlling the adipo-osteogenic lineage commitment of BMSCs in favor of osteogenesis is considered a promising approach for bone regeneration and repair. The exogenous administration of P7C3 in vivo resulted in bone being able to maintain its area, architecture, and mechanical strength despite exposure to IR. While the underlying mechanisms remain unclear, they are potentially primarily driven via the upregulation and downregulation of a series of proteins that together preferentially favor osteogenesis at the expense of adipogenesis, facilitate inflammatory resolution, and suppress RANKL and osteoclastogenesis (Fig. 9). Finally, although unconfirmed, Wnt/β-catenin signaling may be the dominant pathway activated. Preliminarily, and remarkably, at the same protective P7C3 dose, a significant reduction in triple-negative breast cancer and osteosarcoma cell metabolic activity was found in vitro. Together, these results indicate that P7C3 may serve as a novel multifunctional therapeutic strategy for IR-induced bone loss, and further work is needed to investigate this over the longer term post-IR, as well as its ability to selectively drive cancer cell death (Fig. S17).

## Materials and methods

### In vitro analyses

#### Culture and irradiation of cells

Human bone marrow-derived mesenchymal stromal cells (hBMSCs, ATCC^®^ PCS-500-012™), murine-derived macrophages (RAW 264.7 cells, ATCC^®^ TIB-71™), human osteosarcoma cells (hOS; ATCC® CRL-1547™), metastatic prostate cancer cells (PC3; ATCC^®^ CRL-1435™), and triple-negative breast cancer cells (MDA-MB-231/GFP; AKR-201, Cell Biolabs, USA) were investigated in this study. hBMSCs, hOS cells, and MDA-MB-231/GF cells were maintained in Dulbecco’s modified Eagle’s medium (DMEM; Thermo Fisher Scientific, USA) with 10% fetal bovine serum (FBS; Thermo Fisher Scientific, USA) and 1% (v/v) penicillin/streptomycin in a humidified incubator containing 5% CO_2_ at 37 °C. RAW 264.7 cells were maintained in DMEM with 10% heat-inactivated FBS and 1% (v/v) penicillin/streptomycin in a humidified incubator containing 5% CO_2_ at 37 °C. PC3 cells were maintained in ATCC-formulated F-12K medium (30-2004, ATCC) with 10% FBS and 1% (v/v) penicillin/streptomycin in a humidified incubator containing 5% CO_2_ at 37 °C.

P7C3 (500 mg, HY-15976) was purchased from MCE (MedChemExpress, USA). For the in vitro analyses, P7C3 was dissolved in dimethyl sulfoxide (DMSO; Fisher Scientific, USA) at a stock concentration of 10 mmol·L^−1^. For analysis of the protective effect of P7C3 on IR-induced cellular damage, cells were pretreated with 1 μmol·L^−1^ or 10 μmol·L^−1^ P7C3 24 h prior to IR exposure. Cells pretreated with 1 μmol·L^−1^ or 10 μmol·L^−1^ DMSO served as a solvent control. After three rinses with phosphate buffered saline (PBS), cells were subjected to 7 Gy irradiation with a 160 kV tube voltage, 4 mA tube current, and a distance of 30 cm between the source and the surface (SC 500 smart controller, KIMTRON, USA). After IR, the cell media was replenished with either 1 μmol·L^−1^ or 10 μmol·L^−1^ P7C3. Cells in the solvent control group received 1 μmol·L^−1^ or 10 μmol·L^−1^ DMSO.

#### Analysis of P7C3 on the osteoclastic differentiation of murine RAW264.7 macrophages

Following IR-induced damage, RAW264.7 macrophages were cultured in DMEM containing 10% FBS with or without P7C3 in a humidified incubator containing 5% CO_2_ at 37 °C for 1 or 3 days. Cells were fixed with 4% paraformaldehyde for 20 min. Cell morphological characteristics were studied using Alexa Fluor™ 488 Phalloidin and DAPI double staining. Images were captured using confocal laser scanning microscopy (Zeiss, USA), and the cell area (µm^2^) was quantified using ZEN 3.1 software (Zeiss, USA). Additionally, tartrate-resistant alkaline phosphatase (TRAP) staining was used to determine the formation of activated osteoclastic cells from macrophages. Images were captured using an inverted phase microscope (BZ-X800E, Keyence, USA).

#### Analyses of P7C3 on hBMSC and RAW264.7 macrophage metabolic activity and morphology

The toxicity of P7C3 on hBMSCs was determined using a live/dead assay according to the protocol developed by ibidi, USA. After staining, images of live (green) and dead (red) cells were taken using an inverted phase microscope (BZ-X800E, Keyence, USA).

An MTT [3-(4,5-dimethylthiazol-2-yl)-2,5-diphenyl tetrazolium bromide] assay (M2128, Millipore Sigma, USA) was further performed as per our previous study.^133^ Absorbance was measured using a microplate reader (Agilent BioTek Synergy H1 Hybrid Multi-Mode Reader, USA) at 570 nm. Cell number and morphological changes were further evaluated using confocal laser scanning microscopy. Briefly, cells were fixed with 4% paraformaldehyde for 20 min. The actin filaments and nuclei were stained using Alexa Fluor™ 594 Phalloidin (A12381, Thermo Fisher Scientific, USA) or Alexa Fluor™ 488 Phalloidin (A12379, Thermo Fisher Scientific, USA) and 4′,6-diamidino-2-phenylindole dihydrochloride (DAPI; D9542, Millipore Sigma, USA), respectively. Images were captured using confocal laser scanning microscopy (Zeiss, USA), and the cell area (µm^2^) was quantified using ZEN 3.1 software (Zeiss, USA).

#### DNA damage in hBMSCs

A Comet Assay^®^ (4250-050-K, R&D Systems, USA) was used to assess IR-induced DNA damage to hBMSCs. Briefly, the cells were detached gently using a cell scraper, pelleted and then resuspended in ice-cold PBS (Ca^++^ and Mg^++^ free). A 50 µL cell suspension was mixed with 500 µL of molten LMAgarose at 37 °C. Fifty microliters of the mixture was then pipetted immediately onto a CometSlide™. After a 10 min solidification period at 4 °C, the slides were immersed in a prechilled lysis buffer overnight at 4 °C. After cell lysis, slides were immersed in a freshly prepared alkaline unwinding solution for 20 min, followed by 30 min of electrophoresis at 21 volts. The slides were washed twice in deionized H_2_O, followed by washing in 70% ethanol. One hundred microliters of diluted SYBR^™^ Green I Nucleic Acid Gel Stain was used to stain the slides in the dark for 10 min. The slides were visualized using confocal laser scanning microscopy (Zeiss, USA).

#### Osteogenic differentiation of hBMSCs

After P7C3 pretreatment (24 h) and 7 Gy of IR, hBMSCs were cultured in DMEM containing 10% FBS with osteogenic components (2 mmol·L^−1^ β-glycerophosphate, 100 μmol·L^−1^ L-ascorbic acid 2-phosphate, 10 nmol·L^−1^ dexamethasone). The medium was changed every 3 days and replenished with either 10 μmol·L^−1^ P7C3 or solvent control.

#### Alkaline phosphatase staining

An Alkaline Phosphatase Staining (ALP) Kit (K2035-50, BioVision, USA) was used to assess ALP expression at 14 days. Briefly, the cells were stained with ALP staining solution for 30 min. Images were captured using an inverted phase microscope (BZ-X800E, Keyence, USA).

#### Collagen staining

A Sirius Red/Fast Green Collagen Staining Kit (50-152-6960, Fisher Scientific, USA) was used to detect total collagen levels at 14 days. Images were captured using an inverted phase microscope (BZ-X800E, Keyence, USA).

#### Alizarin red S staining

Alizarin red S staining (A5533-25G, Millipore Sigma, USA) was performed to evaluate mineral deposition at 14 and 28 days. Briefly, cells were fixed with 4% paraformaldehyde and stained with 2% pH 4.1 alizarin red S solution for 20 min at room temperature. Images were captured using an inverted phase microscope (BZ-X800E, Keyence, USA). Cetylpyridinium chloride (10%, C0732-100G, Sigma, USA) was used to dissolve the mineral deposition, and the optical density (OD) values were determined at 562 nm (Agilent BioTek Synergy H1 Hybrid Multi-Mode Reader, USA).

#### Analysis of IR-induced cellular senescence in hBMSCs

Cellular senescence in hBMSCs was detected using a senescence-associated β-gal (SA-β-gal) staining kit (9860, Cell Signaling Technology, USA) after 28 days of culture, as per our previous study.^55^

#### Adipogenic differentiation of hBMSCs

After P7C3 pretreatment (24 h) and 7 Gy of IR, hBMSCs were cultured in a MesenCult™ Adipogenic Differentiation Kit (05412, STEMCELL Technologies, USA). The medium was changed every 3 days and replenished with either P7C3 or solvent control. Gene expression of adipogenic-related marker genes in hBMSCs was examined via qRT‒PCR using the methods described above.

#### Lipid droplet staining

At 14 and 21 days, cells were fixed with 4% paraformaldehyde and stained with LipidSpot™ Lipid Droplet Stains (Biotum, Inc., Fremont CA, USA) according to the manufacturer’s protocol. Images of stained sections were captured using an inverted phase microscope (BZ-X800E, Keyence, USA).

#### Quantitative real-time reverse-transcription polymerase chain reaction (qRT‒PCR)

Gene expression of adipogenic-related marker genes in hBMSCs (*ADPOQ, PPARG, LPL, LEP, FABP4, CEBPA, PLIN1*, and *FASN*) was examined via qRT‒PCR at 12 days. Briefly, total RNA was extracted using a PureLink™ RNA Mini Kit (12183018 A, Thermo Fisher Scientific, USA), and genomic DNA contamination was removed from samples using DNase (PureLink™ DNase Set, 12185010, Thermo Fisher Scientific, USA). RNA concentration was measured using a NanoDrop 8000 spectrophotometer (NanoDrop Technologies). Five hundred nanograms of total RNA was used as a template for reverse transcription, and cDNA synthesis was performed using SuperScript™ III First-Strand Synthesis SuperMix (18080400, Thermo Fisher Scientific, USA). qRT‒PCR was performed using Fast SYBR™ Green Master Mix (4385612, Thermo Fisher Scientific, USA) on an ABI Prism 7500 Thermal Cycler (Applied Biosystems, Foster City, California, USA). The primers used in this study were KiCqStart™ Primers and were purchased from Millipore Sigma. The fold change in relative mRNA expression was calculated using the comparative Ct (2^−ΔΔCT^) method.

### In vivo analyses

#### Exposure of the rat hind limb to IR and P7C3 treatment

Eighteen male SAS Sprague Dawley rats, 8–9 weeks of age (~200 g), were investigated in vivo. Animals were acclimatized for 1 week before commencing the experiment. All procedures involving animals were approved by the Institutional Animal Care and Use Committee at the University of Central Florida (protocol 2020-48; approved most recently in July 2022) and were performed in accordance with the American Veterinary Medical Associated guidelines. Rats were randomly divided into three groups (*n* = 6): control, X-ray, and X-ray + 20 mg·kg^−1^ P7C3. The main body of the rat was shielded for protection using a lead blanket (MPS-S, Z&Z Medical, USA), and the hind limbs (only) were subjected to a local fractionated X-ray dose of 8 Gy on Days 1, 3, and 5 of the study (a total dose of 24 Gy). X-ray exposure was applied at a tube voltage of 160 kV, a 4 mA tube current, and at a distance of 30 cm between the source and the surface of the animal (SC 500 smart controller, KIMTRON, USA). This biological irradiator produces a polyenergetic beam similar to the irradiators used clinically. Animal positioning, lead shielding during exposure (all areas shielded except the hind limbs), dose rate, distance from the source, and radiation intensity were kept consistent. P7C3 (20 mg·kg^−1^) was administered as a pretreatment prior to IR and then daily via intraperitoneal (*i.p*.) injection until the end of the study (7 days).

### Histological staining

#### Whole-mount histological staining for bony structure

Briefly, harvested bone was fixed in 10% neutral buffered formalin, dehydrated in graded ethanol, and embedded in paraffin wax. Longitudinal sections were prepared through the distal femoral condyle. Briefly, 5 μmol·L^−1^ thick sections were prepared from the paraffin-embedded samples, followed by deparaffinizing and rehydrating. A hematoxylin and eosin (H&E) staining kit was used to detect the bony structure (ab245880, Abcam, USA). Images of stained sections were captured using an inverted phase microscope (BZ-X800E, Keyence, USA).

#### TRAP staining for osteoclastic activity

TRAP staining was used to detect osteoclastic activity using the standard naphthol AS-BI phosphate postcoupling method. Paraffin sections (5 μmol·L^−1^) were rehydrated and incubated with TRAP staining solution containing 0.2 mol·L^−1^ sodium acetate buffer (pH 5.0, S2889-250G, Millipore Sigma, USA), 50 mmol·L^−1^ L-(+) tartaric acid (228729-100 G, Millipore Sigma, USA), 0.5 mg·mL^−1^ naphthol AS-MX phosphate (N4875-100MG, Millipore Sigma, USA), and 1.1 mg·mL^−1^ Fast Red TR salt (ab146351, Abcam, USA) for 2 h at 37 °C. Nuclei were then counterstained with hematoxylin (ab245880, Abcam, USA) for 5 min before mounting with VectaMount™ AQ Mounting Medium (Vector Laboratories, USA). Slides were visualized using an inverted phase microscope (BZ-X800E, Keyence, USA).

#### Nano-CT scanning

Tibiae were prepared for high-resolution X-ray computed tomography (CT-scanning) using a GE V|TOME|X M 240 Nano CT scanner (General Electric; University of Florida, Gainesville, FL, USA). Samples were scanned using a 180 kv X-ray tube with a diamond-tungsten target under the following settings: 75 kV, 150 mA, a 0.5 second detector time, averaging of three images per rotation and a voxel resolution of 12.4 μmol·L^−1^. Three-dimensional models of the trabecular network within the proximal tibia were created using a 3D Slicer™ (v4.11.20210226; Brigham and Women’s Hospital and Massachusetts Institute of Technology). The DICOM files were imported, and a label map was created. A threshold was used to automate the segmentation process, and a smooth crushing tool was used to manually clean the segments.

#### Analysis of bone strength via 3-point bending

Immediately after dissection, the retrieved tibiae were plastic wrapped and stored at −20 °C. Samples were thawed at room temperature, and 3-point bending analyses were carried out within 96 h of tissue retrieval. Each tibia was positioned horizontally on support bars positioned 8 mm apart. With the tibial anterior bow facing downward and using a universal testing machine (Criterion^®^ 43, MTS, Eden Prairie, MN, USA), a vertical force was applied to the tibial mid-shaft using a 3 mm diameter leading roller and a 5 kN load cell. Each tibia was loaded until failure at a displacement rate of 0.02 mm·s^−1^. As the cross-sectional area of the tibia was nonuniform and similar to other studies,^134–136^ we assumed that the cross-sectional area was circular and obtained the mechanical properties from the load‒displacement curve and using the following formulae:

1 $${\rm{\sigma }}\,=\,\frac{F* L* {c}_{O}}{4* I}$$

2 $$E=\,\frac{F{L}^{3}}{d* 48* I}$$

where *σ* is the stress (Pa), F is the applied load (N), L = 0.008 is the span distance between the supports (m), ${c}_{o}$ is the outer radius of the tibial midshaft (m), which was measured using a caliper (Digital, Cole-Parmer, IL, US), E is the elastic modulus (Pa), d is displacement (m), and I is the moment of inertia (m^4^) calculated as follows:

3 $$I=\,\frac{\pi }{4\left({c}_{o}^{4}-{c}_{i}^{4}\right)}$$

where ${c}_{i}$ is the inner radius of the tibial midshaft (m). The inner radius of the midshaft was measured from the μCT scans. Four cross-sections *per* bone and from each experimental group were selected, and both inner and outer diameters were calculated from each cross-section. The mean value of the inner-to-outer diameter from each cross-section was calculated to determine the inner radius. The ultimate stress (σu) and fracture stress (σf) were calculated. The mechanical strength parameters were adjusted for body size (ratio of body weight to tibial length).^137^

#### Immunohistochemistry staining

Immunohistochemistry (IHC) staining was performed according to the Abcam protocol (https://www.abcam.com/ps/pdf/protocols/ihc_p.pdf). Briefly, sections (5 μmol·L^−1^) were deparaffinized in xylene and hydrated in descending alcohol and deionized H_2_O before immersion in an antigen retrieval buffer. Antigen retrieval was performed by boiling slides in 10 mmol·L^−1^ sodium citrate buffer (pH 6.0). Endogenous peroxidase activity was inactivated in a hydrogen peroxide blocking reagent (ab64218, Abcam, USA) for 10 min. After PBS washes, sections were blocked using a protein block (ab64226, Abcam, USA) at room temperature for 30 min. The following antibodies were used as primary antibodies overnight incubation: RANKL (NB100-56512, Novus Biologicals, USA) and perilipin (NB110-40760, Novus Biologicals, USA). Goat anti-mouse IgG secondary antibody [HRP Polymer] (VC001-025, Novus Biologicals, USA) or goat anti-rabbit mouse IgG secondary antibody [HRP Polymer] (VC002-025, Novus Biologicals, USA) was used as secondary antibodies. Samples were stained with the SignalStain^®^ DAB Substrate Kit (#8059; Cell Signaling Technology, USA) and counterstained with hematoxylin for 3 min. All stained sections were dehydrated in ascending alcohol baths, cleared in xylene, and coverslipped using Fisher Chemical™ Permount™ Mounting Medium (SP15-100, Fisher Scientific, USA). Images were captured using an inverted phase microscope (BZ-X800E, Keyence, USA).

#### Rat cytokine array

Blood was obtained from each animal following cardiac puncture. Blood samples were collected in blood collection tubes (SST-Serum Separator Tube, IDEXX BioAnalytics, USA), and sera were collected for cytokine analysis (Rat L2 Array, Glass Slide; AAR-BLG-2-4, RayBiotech Life, GA, USA). The signal intensities of 500 protein targets were plotted as a heatmap in which the different colors represent biomarker expression levels. The targets were then subjected to hierarchical clustering based on Euclidean distance. Gene Ontology (GO) term enrichment and Kyoto Encyclopedia of Genes and Genomes (KEGG) pathway enrichment analyses were conducted as overrepresentation evaluations of the differentially expressed biomarkers involved in the various KEGG pathway/GO terms. All of the targets measured formed the background. All analyses were conducted in the R programming language V3.6.3 (R Core Team 2017). Pathway/GO overrepresentation was implemented using the R package clusterProfiler.^138^

### Bone marrow adiposity

#### Sudan black B staining

Five-micron-thick sections were prepared from paraffin-embedded samples, followed by deparaffinizing and rehydrating. Then, the tissue sections were stained with Sudan black B solution (4197-25-5, Millipore Sigma, USA) for 3 h. After rinsing with 70% isopropyl alcohol and distilled water, nuclear fast red (50-317-51, Electron Microscopy Sciences, USA) was used for counterstaining. Images were captured using an inverted phase microscope (BZ-X800E, Keyence, USA).

#### Oil red O staining

Frozen tissue sections (20 μmol·L^−1^) were used for Oil red O staining (O1391-250ML, Millipore Sigma, USA). Briefly, slides were rinsed with PBS and 60% isopropanol and stained with Oil Red O solution for 15 min. Hematoxylin was used for counterstaining. Images were captured using an inverted phase microscope (BZ-X800E, Keyence, USA).

#### Cellular senescence

Frozen sections (20 μmol·L^−1^) were prepared for the detection of *β*-gal activity using the methods described above. Nuclear fast red (50-317-51, Electron Microscopy Sciences, USA) was used for counterstaining. Images were captured using an inverted phase microscope (BZ-X800E, Keyence, USA). TNF-α IHC staining was performed according to the methods described above. TNF-α (NBP1-19532, Novus Biologicals, USA) was used as the primary antibody, and goat anti-rabbit mouse IgG secondary antibody [HRP Polymer] (VC002-025, Novus Biologicals, USA) was used as the secondary antibody. Anti-p21/CIP1/CDKN1A antibody (NB1001941) was purchased from Novus Biologicals, USA. Anti-CDKN2A/p16INK4a antibody (ab54210) was purchased from Abcam, USA.

#### IR and P7C3 treatment on cancer cell activity

To investigate the effect of P7C3 and IR on the cancer (MDA-MB-231/GFP, OS and PC3) cell response, we applied (1.) P7C3 treatment only or (2.) IR + P7C3. Cells were treated with P7C3 (10, 20 or 40 μmol·L^−1^) or solvent control for 1 or 3 days. Cells were subjected to irradiation as described above. After X-ray exposure, the cell medium was replenished with P7C3. A cell proliferation assay and morphological analysis were performed using the MTT assay or phalloidin and DAPI staining as described above. A 3D MDA-MD-231 spheroid culture was performed according to the Thermo Fisher protocol (https://www.thermofisher.com/us/en/home/references/protocols/cell-culture/3-d-cell-culture-protocol/mda-mb-231-cell-line-spheroid-generation.html). Spheroids were imaged using confocal microscopy, and spheroid size (µm^2^) was quantified using ZEN 3.1 software (Zeiss, USA) after 14 days of culture.

### Statistical analysis

All assays are presented as the mean ± standard deviation (SD). Statistical analysis was carried out using GraphPad Prism (version 8.0, US), and groups were compared using the nonparametric Mann‒Whitney test. *P* < 0.05 were considered significant.

## Supplementary information

Supplementary Figures

## Data Availability

The data that support the findings of this study are available from the corresponding author, [MC], upon reasonable request.
