# Supplementary material for: A novel multifunctional radioprotective strategy using P7C3 as a countermeasure against ionizing radiation-induced bone loss
Source: Bone Res. 2023 Jun 29;11:34. doi: 10.1038/s41413-023-00273-w (PMC10310858; doi:10.1038/s41413-023-00273-w)
Supplement: Supplementary file 1 — Supplementary Figures [file 41413_2023_273_MOESM1_ESM.docx]

**Supplementary Information**

**A novel multifunctional radioprotective strategy using P7C3 as a countermeasure against ionizing radiation-induced bone loss**

Fei Wei^1^, Zewen Kelvin Tuong^2,3^, Mahmoud Omer^1^, Christopher Ngo^1^, Jackson Asiatico^4^, Michael Kinzel^4^, Abinaya Sindu Pugazhendhi^1^, Annette Khaled^5^, Ranajay Ghosh^4^, Melanie Coathup^1^

**Supplementary Figure S1**

**
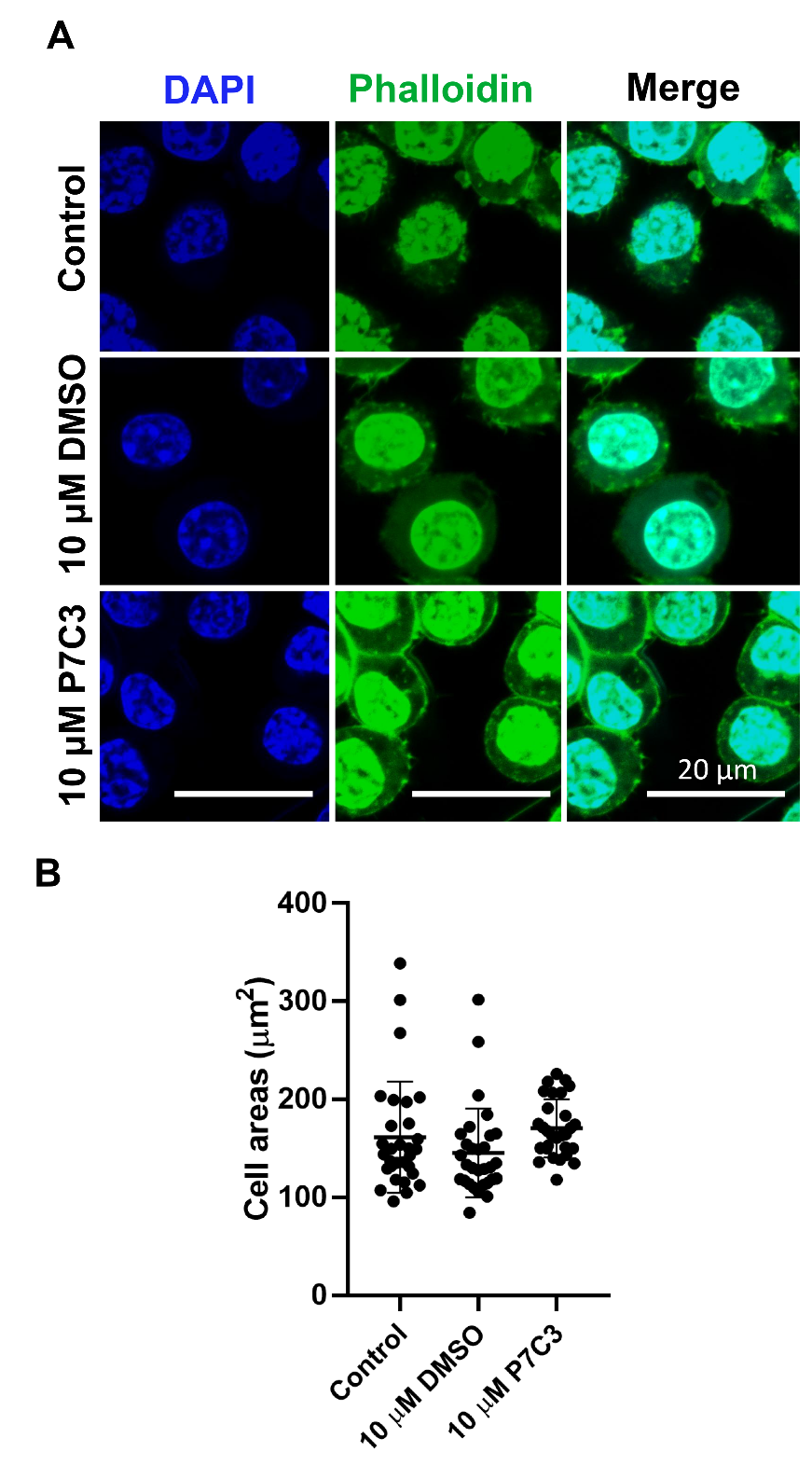
**

**Supplementary Figure S1. Morphological characterization of RAW264.7 macrophages following P7C3 treatment under non-irradiated conditions.** Macrophage morphology was assessed *via* phalloidin (green, actin filaments) and DAPI (blue, cell nuclei) staining. [A] Representative confocal micrographs of RAW264.7 cells after P7C3 treatment at day 1. [B] Quantification of cell area (μm^2^) in each group. Cell area showed no significant differences among groups.

**Supplementary Figure S2**

**
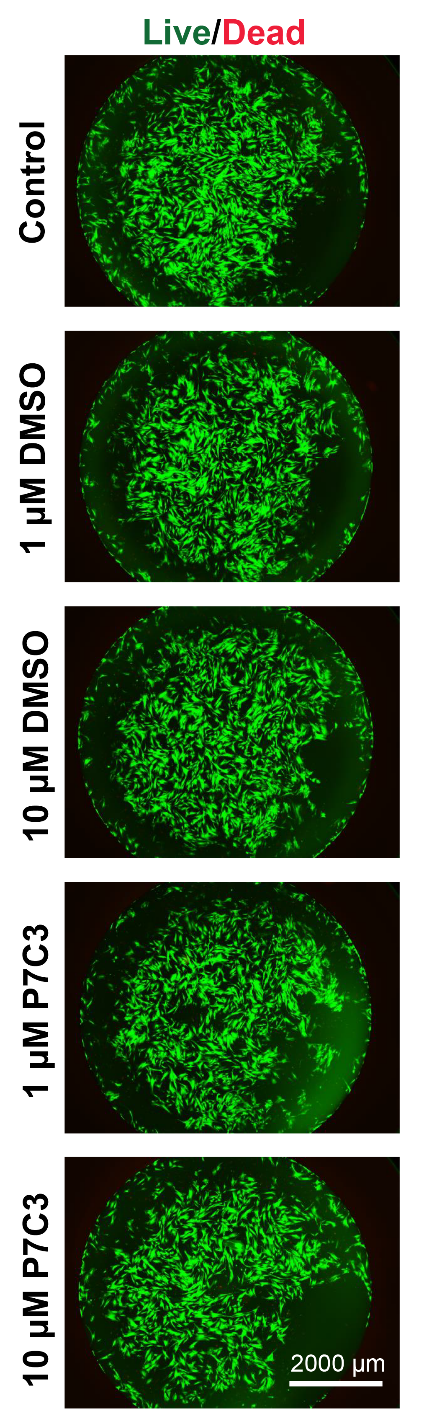
**

**Supplementary Figure S2. Representative confocal micrographs of hBMSCs cultured with increasing concentrations of DMSO and P7C3 for 3 days under non-irradiated conditions**. Cells were double stained with fluorescein diacetate (Live cells, green) and propidium iodide (Dead cells, red), prior to examination using confocal laser scanning microscopy. The results indicate treatment with both concentrations (1 µM or 10 µM) of DMSO and P7C3 showed no apparent changes in the number of live/dead cells when compared with the untreated control cells.

**Supplementary Figure S3**

**
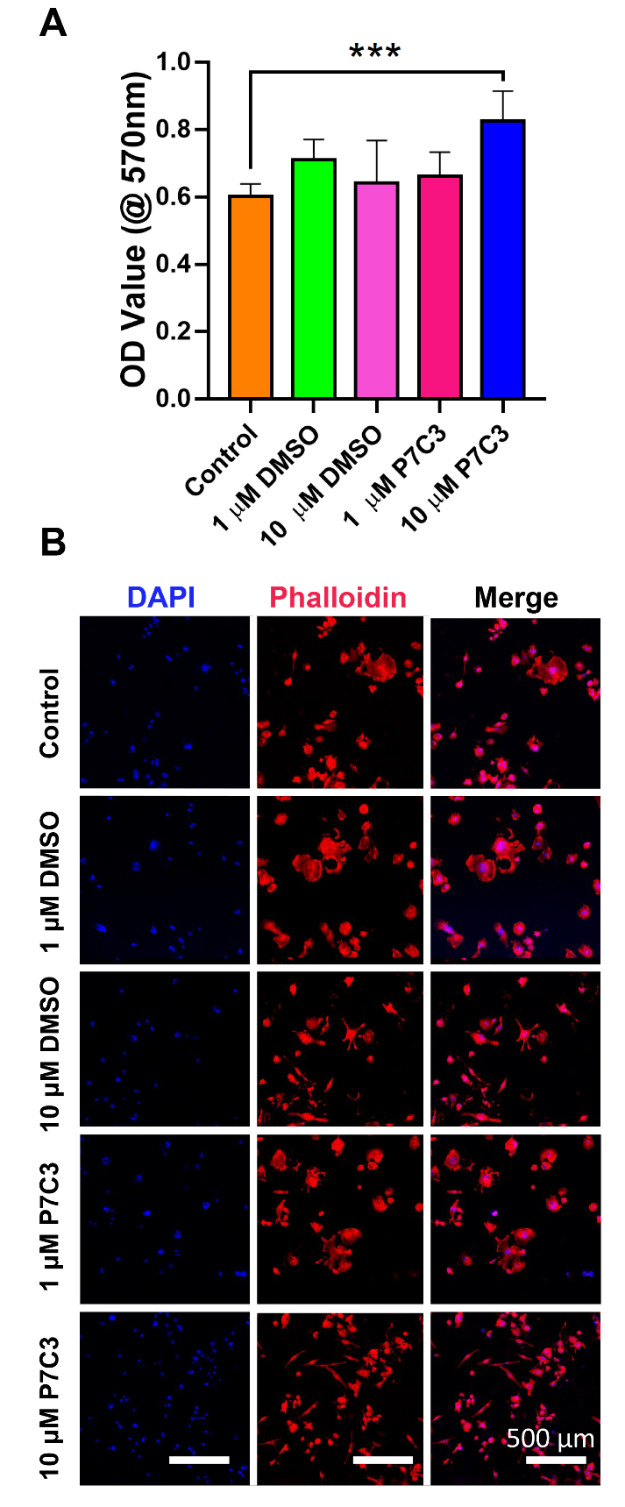
**

**Supplementary Figure S3. Effect of P7C3 on RAW 264.7 macrophage cell metabolic activity and morphology on day 3 following IR.** [A] Cell metabolic activity was determined in X-ray exposed RAW264.7 cells. Using a MTT assay, metabolic activity was quantified and P7C3 treatment significantly increased macrophage activity. [B] Representative confocal micrographs of RAW264.7 treated with either DMSO (1 or 10 μM) or P7C3 (1 or 10 μM) following X-ray exposure. Images were taken at 3-day post-irradiation.


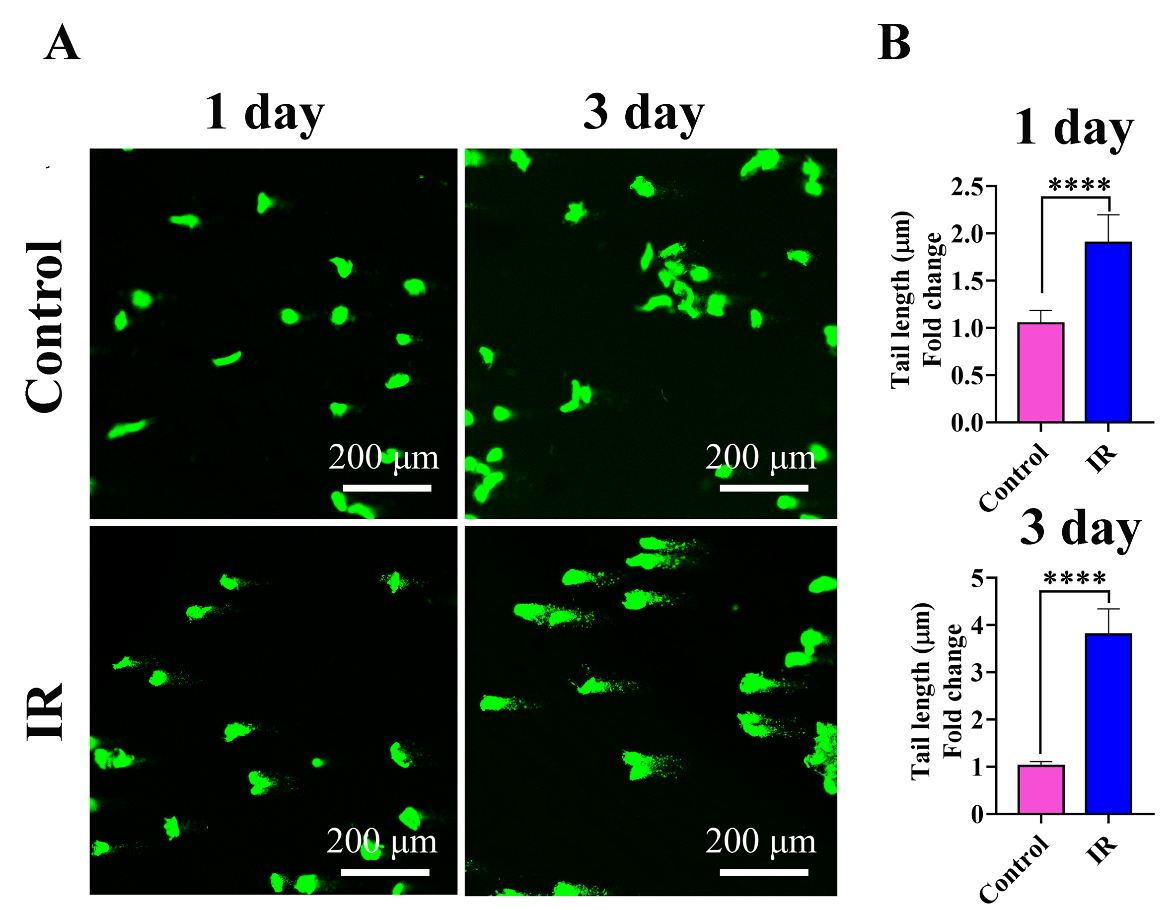
**Supplementary Figure S4**

### Supplementary Figure S4. IR-induced DNA damage in hBMSCs. [A] Representative DNA fragmentation images at 1 day and 3 days in control and IR groups. Images were captured using a confocal laser scanning microscopy. [B] Quantitative analysis revealed that more pronounced DNA damage occurred at day-3 post-IR treatment (>3.8-fold increase) when compared with the day-1 IR group (>1.9-fold increase). *****p* < 0.0001.

**Supplementary Figure S5**


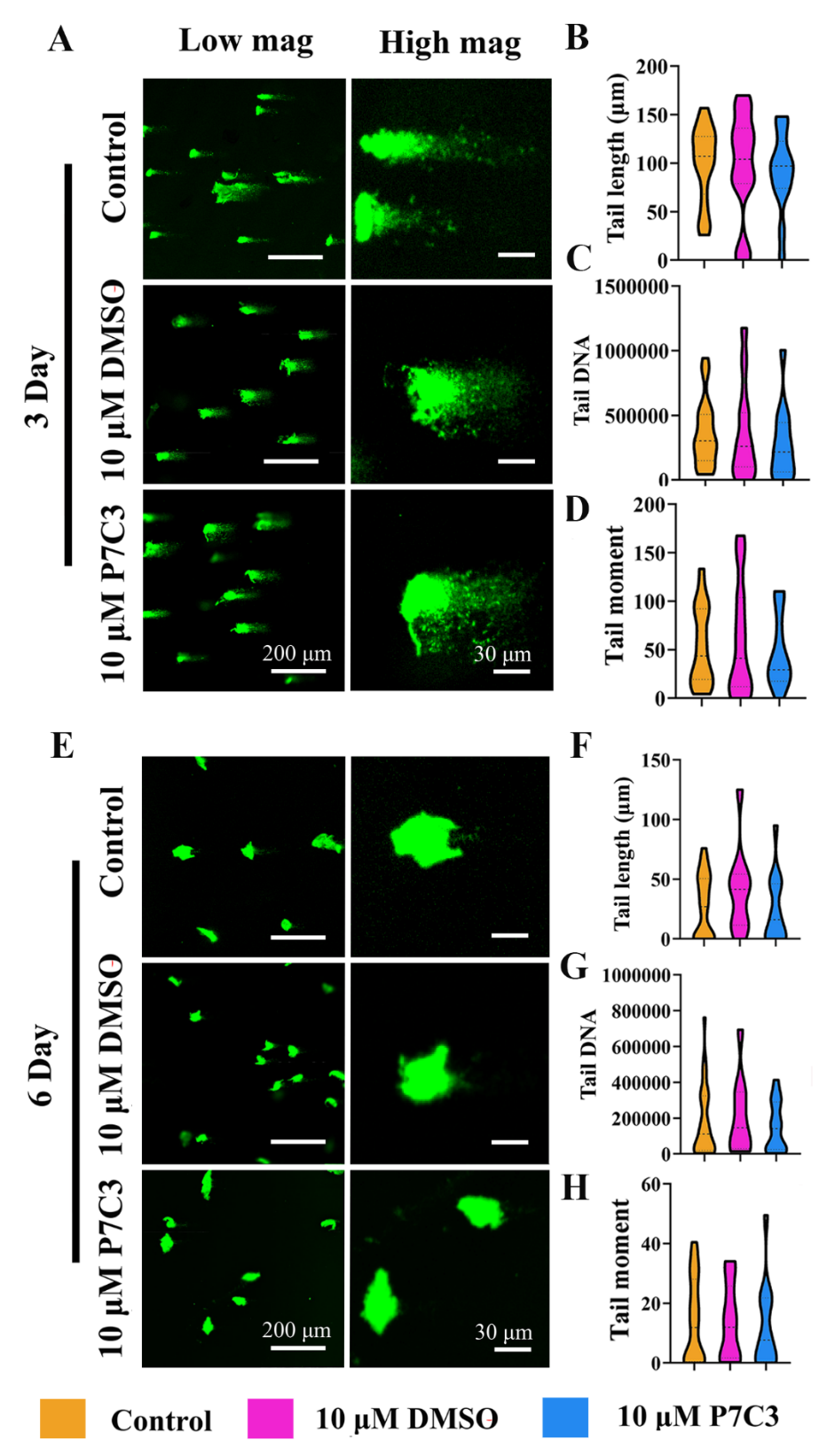


### Supplementary Figure S5. Effect of P7C3 on IR-induced DNA damage in hBMSCs. [A] Representative DNA fragmentation images 3 days after IR. Images were captured using confocal laser scanning microscopy. [B-D] Quantification of tail length, tail DNA, and tail moment at 3 days post-IR. [E] Representative confocal micrographs for DNA damage at 6 days. [F-H] Quantification of tail length, tail DNA, and tail moment at 6 days post-IR. No significant differences were measured.

**Supplementary Figure S6**

**
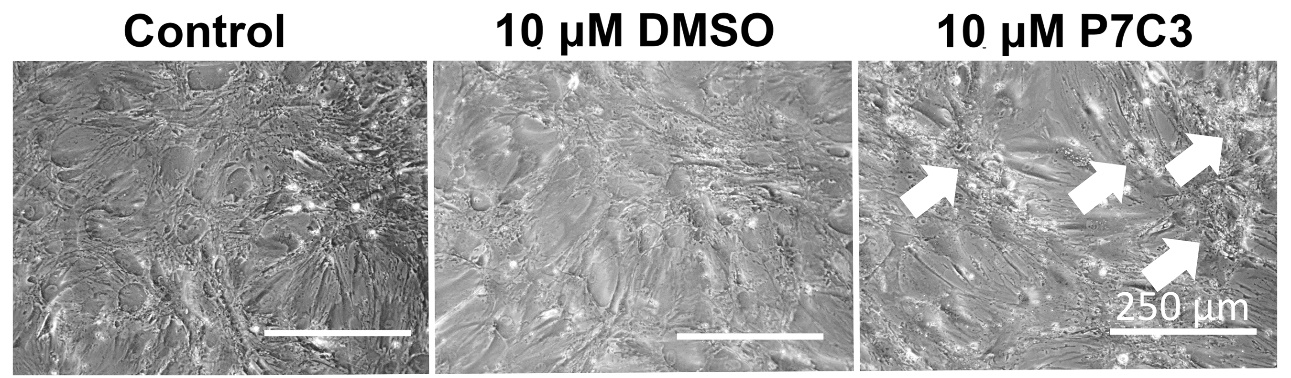
**

**Supplementary Figure S6.** Representative phase contrast images of hBMSCs cultured in osteogenic medium supplemented with 10 μM DMSO (solvent control) or 10 μM P7C3 at 14 days. hBMSCs were pre-treated with either 0, or 10 μM of P7C3 for 24 h before IR. After X-ray, cells were cultured in osteogenic medium supplemented with 10 μM P7C3. 10 μM of DMSO was used as solvent control. Arrows indicate areas of mineralization.

**Supplementary Figure S7**

**
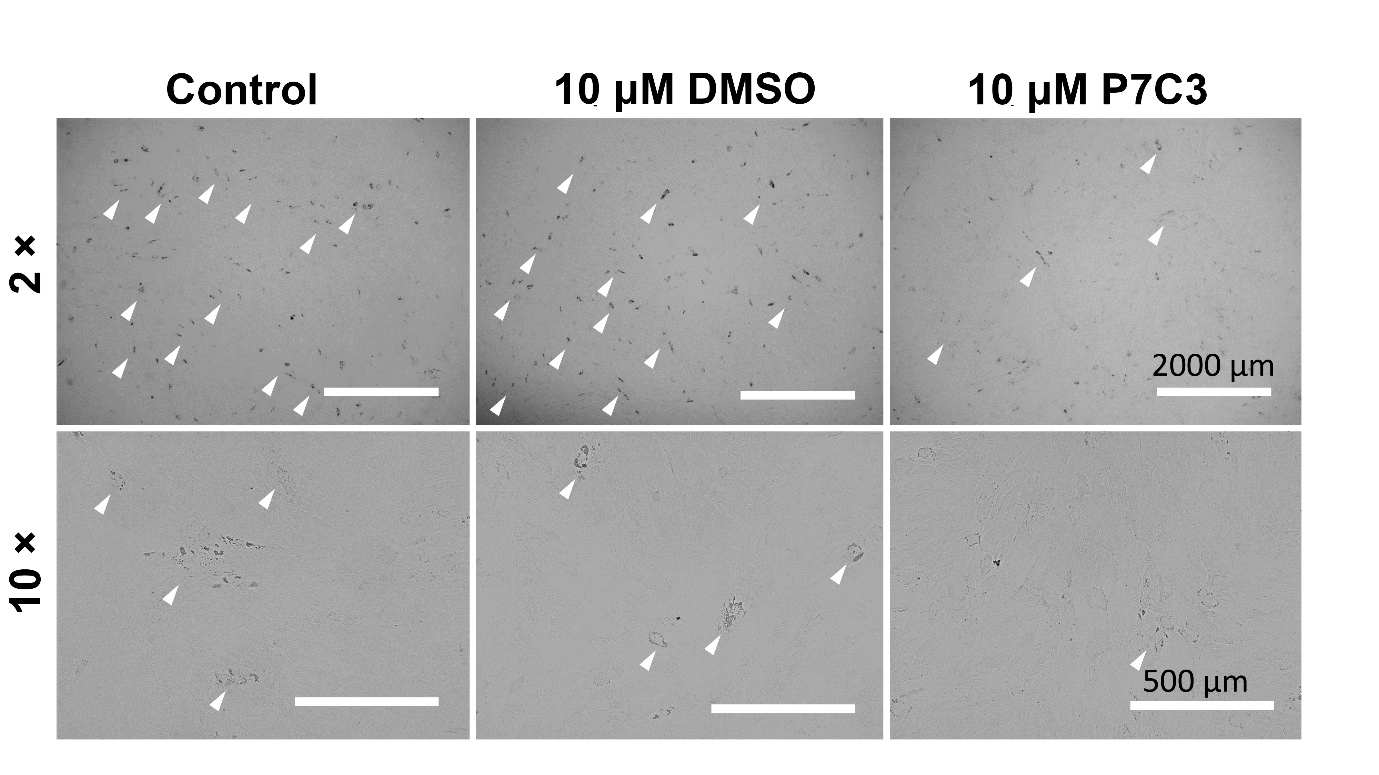
**

**Supplementary Figure S7.** Representative phase contrast images of hBMSCs cultured in adipogenic induction medium supplemented with 10 μM DMSO (solvent control) or 10 μM P7C3 at 12 days. hBMSCs were either pre-treated with 10 μM DMSO or 10 μM of P7C3 for 24 h before IR. After IR, cells were cultured in adipogenic induction medium supplemented with 10 μM DMSO or 10 μM P7C3, respectively. Arrows indicate adipocyte formation.

**Supplementary Figure S8**

**
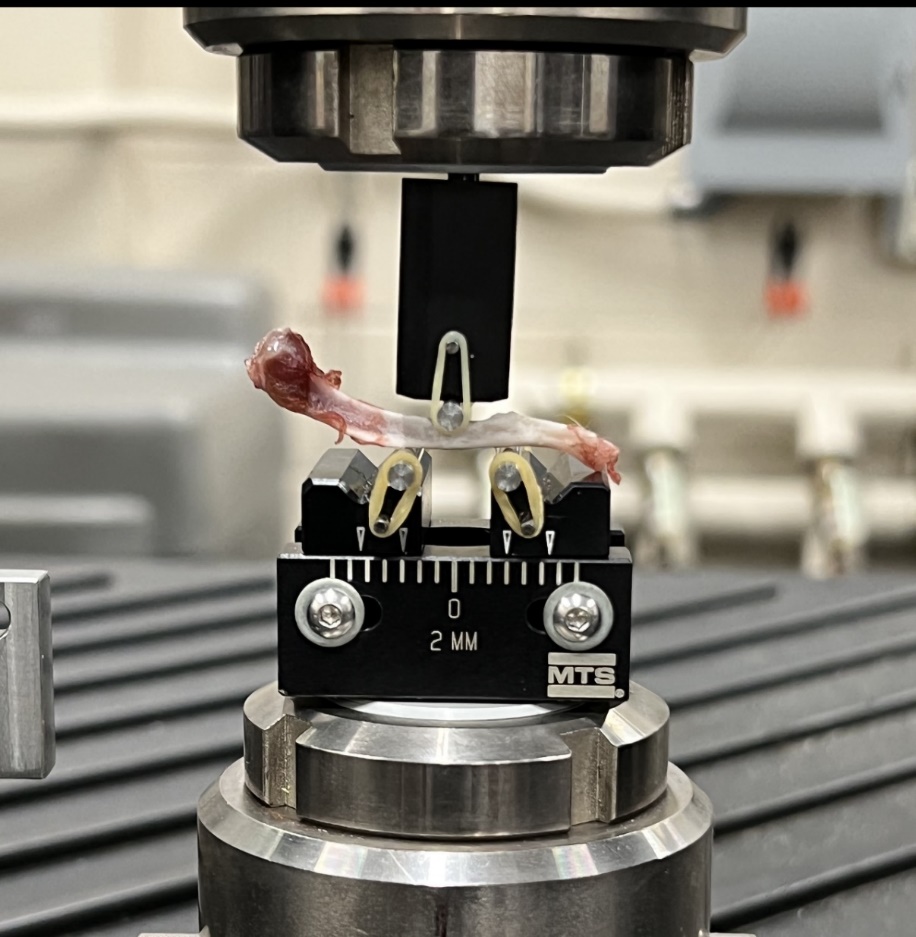
**

**Supplementary Figure S8. The 3-point bending test fixture.** Each tibia was positioned horizontally and using a universal testing machine (Criterion^®^ 43, MTS, Minnesota, USA), a vertical force was applied to the mid-shaft of the retrieved tibiae until complete failure using 8 mm diameter loading roller at a displacement rate of 0.02 mm/s.

**Supplementary Figure S9**

**
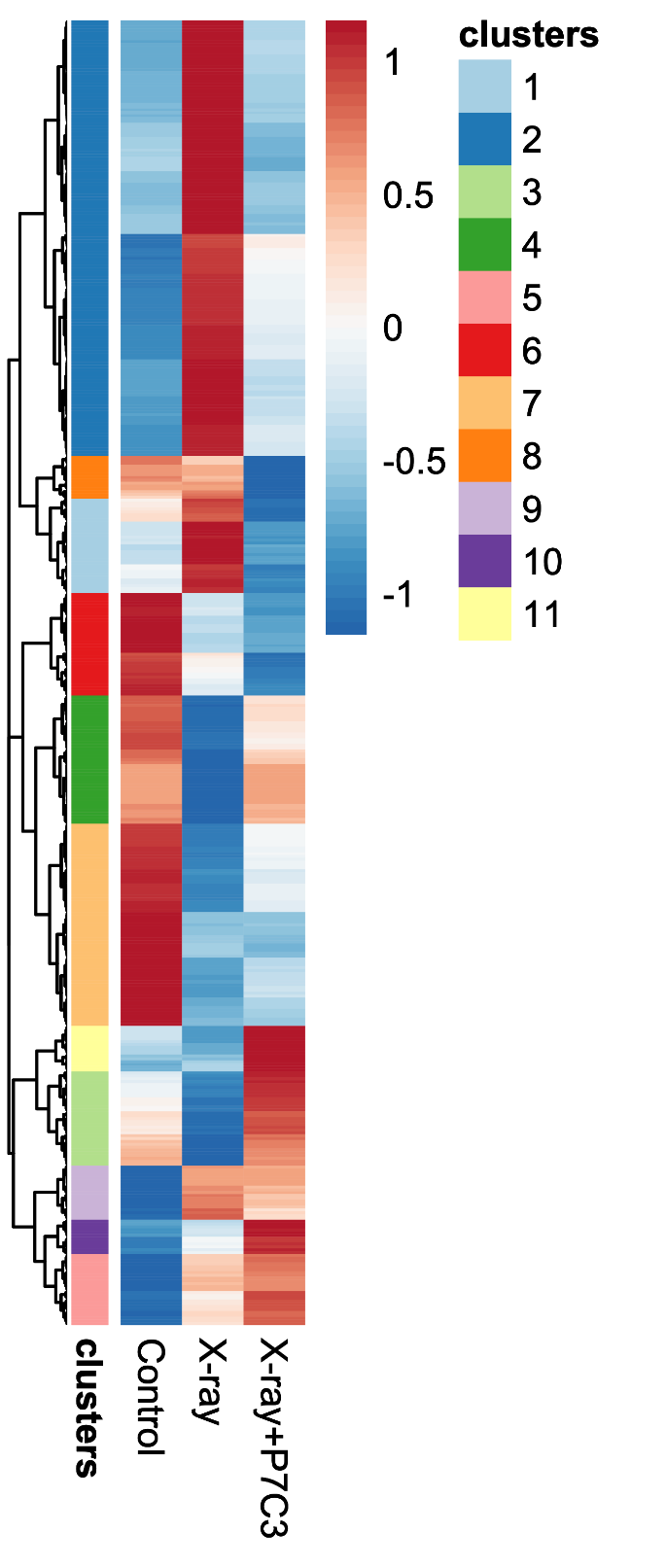
**

**Supplementary Figure S9. Hierarchical clustering of the 500 biomarkers.** The 500 biomarkers were clustered into 11 groups.

**
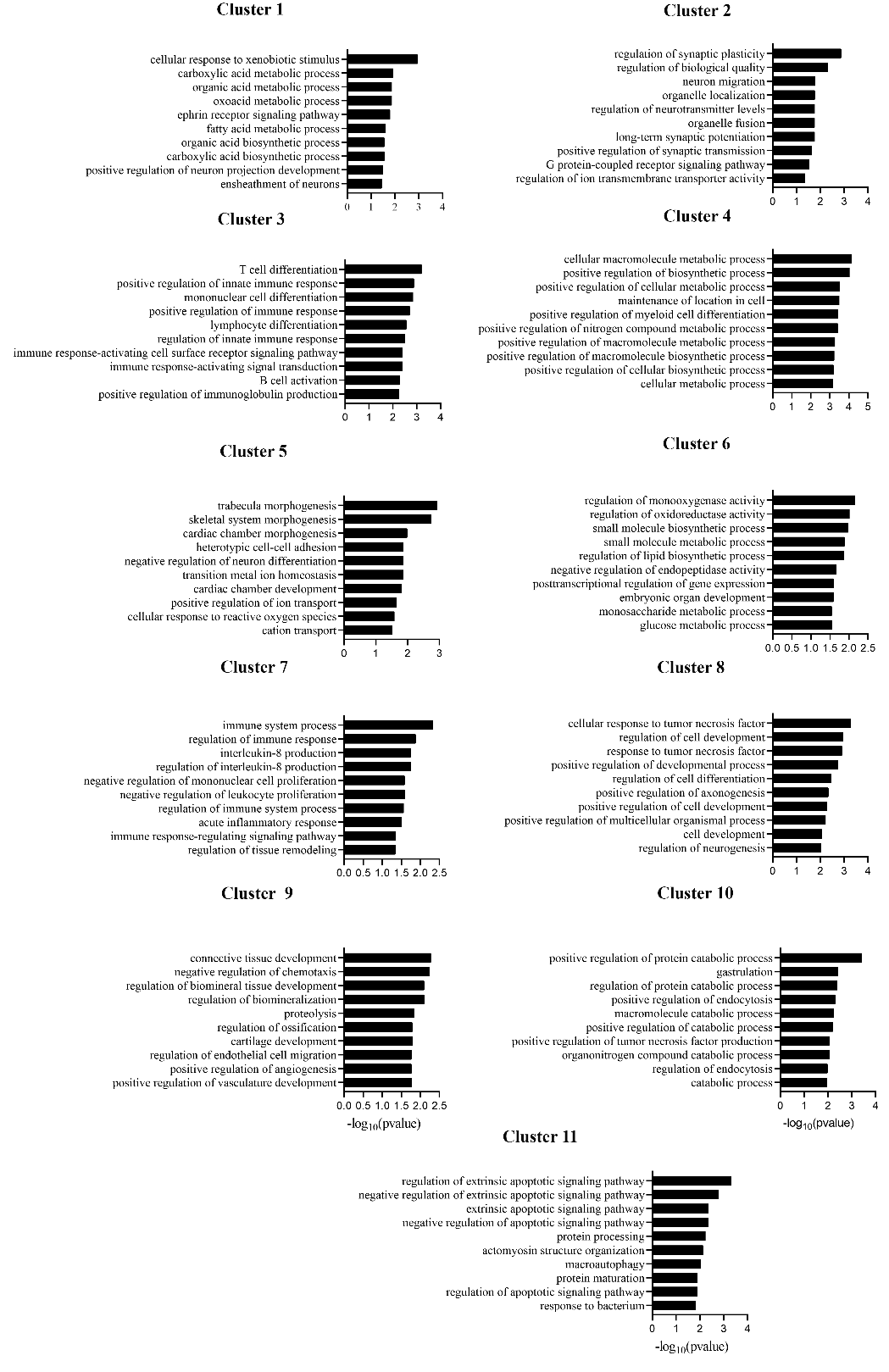
Supplementary Figure S10**

**Supplementary Figure S10.** GO enrichment analysis of each cluster. The enriched GO terms of differentially expressed proteins in each of the 11 clusters.

**
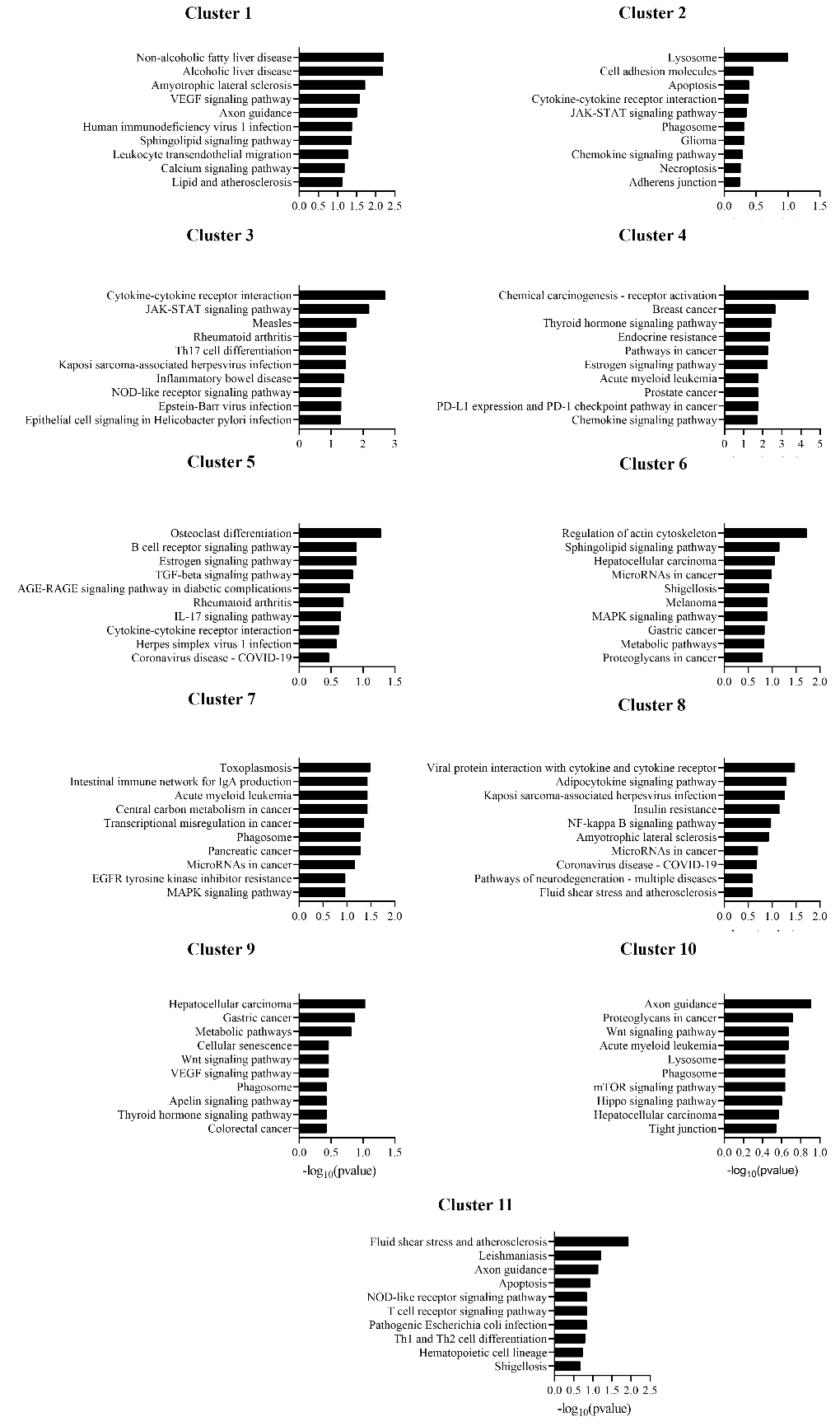
Supplementary Figure S11**

**Supplementary Figure S11. KEGG pathway analysis of each cluster.** The KEGG pathway analysis of differentially expressed proteins in each cluster.

**Supplementary Figure S12**

**
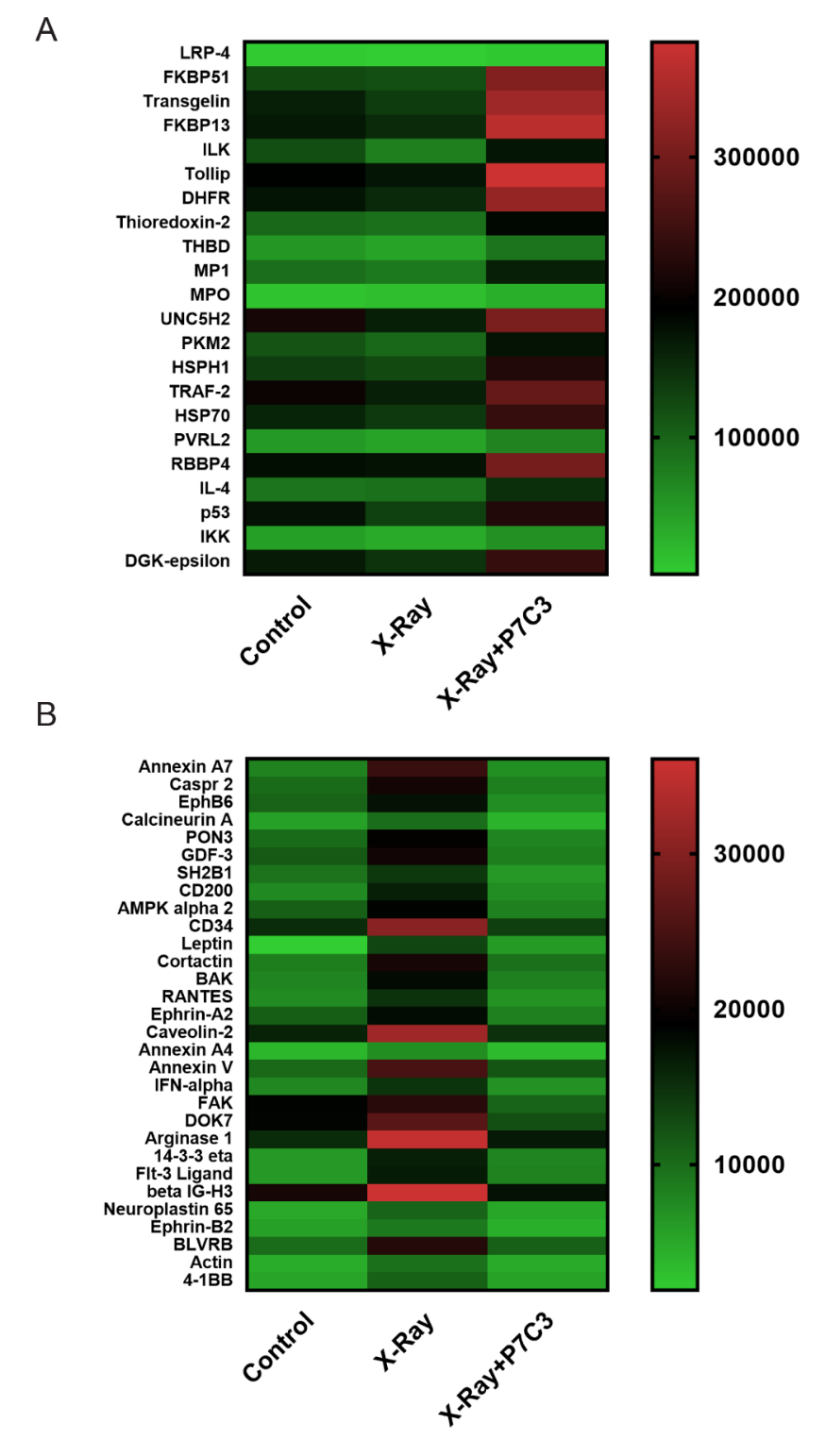
**

**Supplementary Figure S12. Heatmaps of the cytokine profile in plasma following P7C3 treatment.** [A] The expression levels of the top 30 differentially expressed targets (upregulated) were plotted across control, IR, and IR+P7C3 groups. [B] A heatmap of normalized expression levels of the top 30 differentially expressed proteins (downregulated) following P7C3 treatment are presented. The signal-intensities of targets were plotted as a heatmap in which the different colors represent biomarker expression levels.

**Supplementary Figure S13**


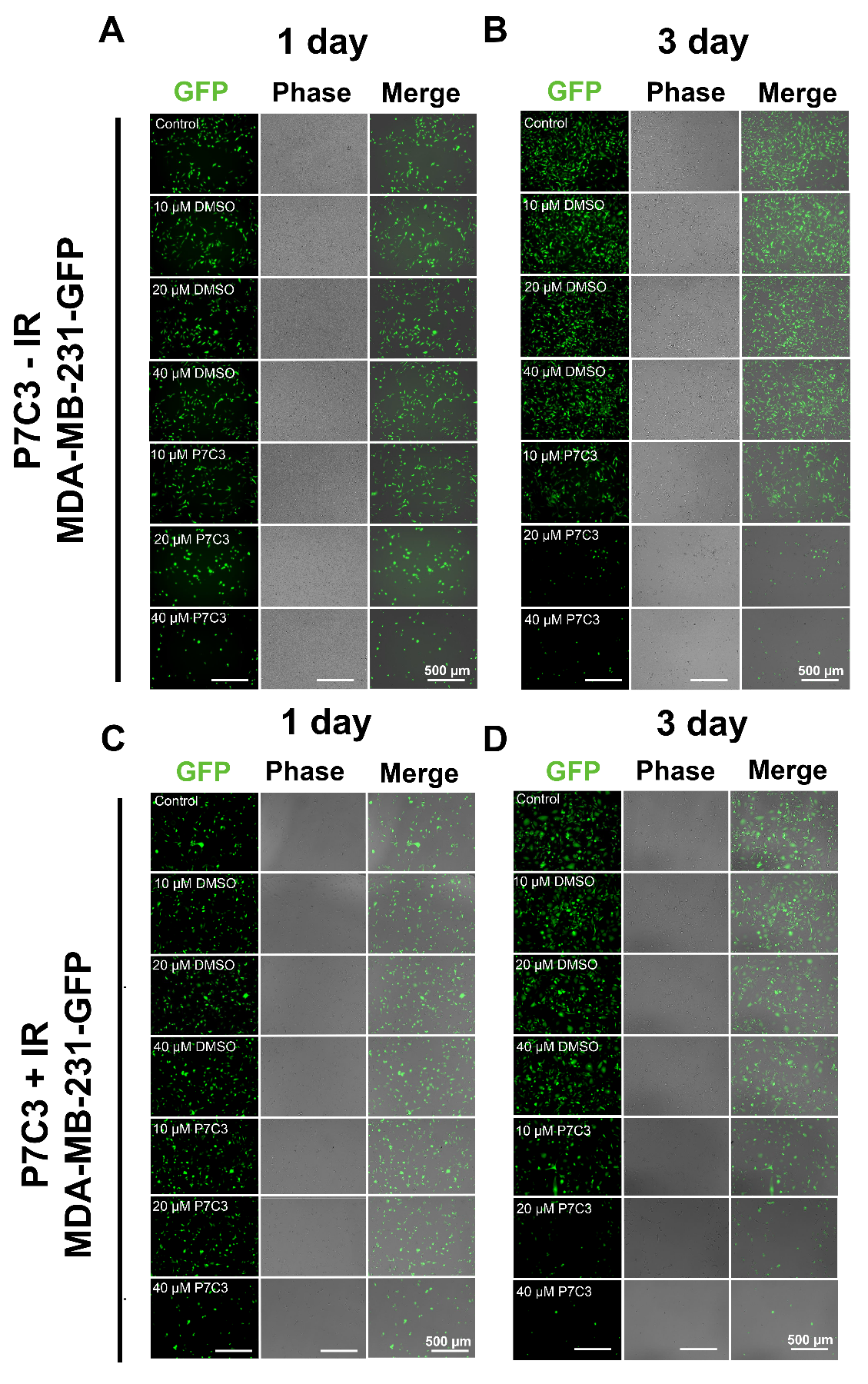


**Supplementary Figure S13. Exposure of MDA-MB-231-GFP cells to P7C3 ± IR decreases cell metabolic activity in a dose-dependent manner.** [A-D] Representative confocal micrographs of MDA-MB-231-GFP cells at 1 day and 3 days post P7C3 ± IR exposure. The MDA-MB-231-GFP cells were incubated with increasing concentrations of P7C3 or solvent control ± IR. Images were acquired *via* live-cell imaging using confocal laser scanning microscopy.

**Supplementary Figure S14**


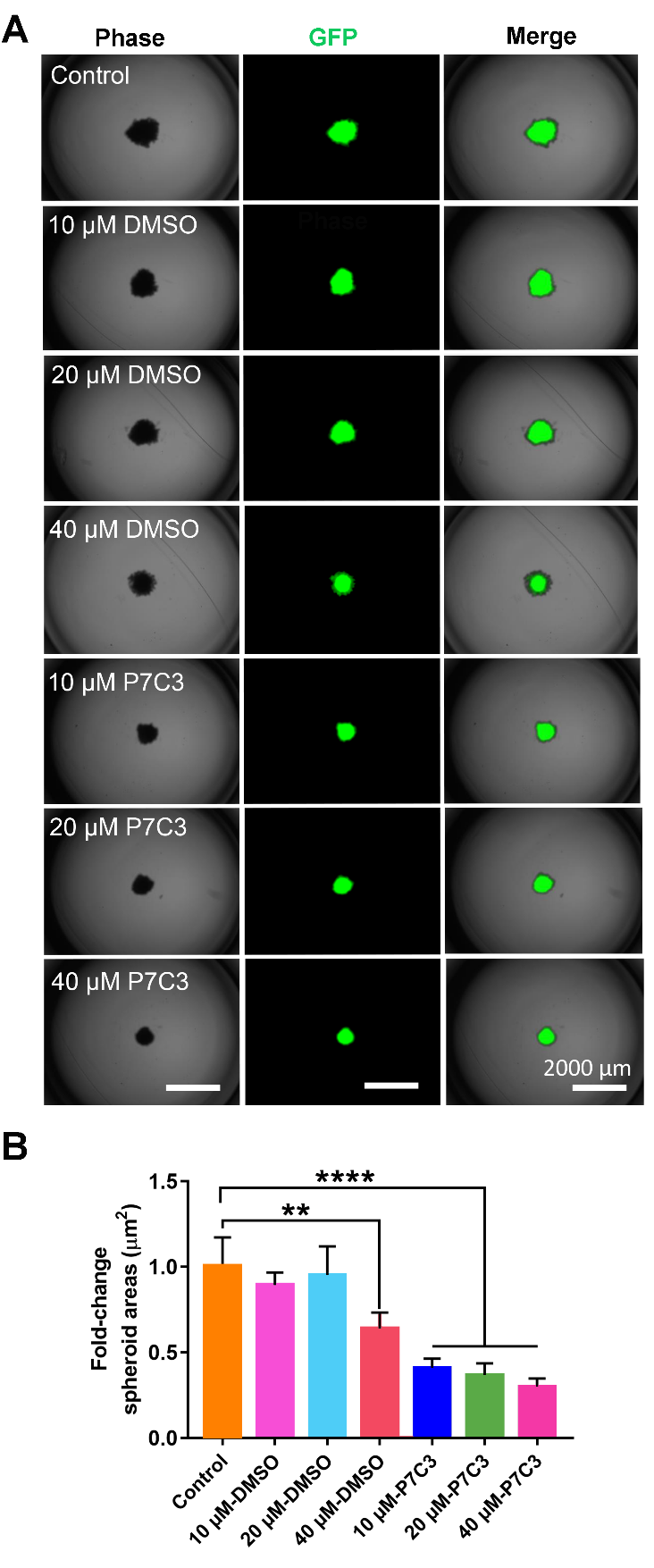


**Supplementary Figure S14. Exposure of MDA-MB-231-GFP spheroids to P7C3 significantly decreases spheroid size** [A] Representative confocal micrographs of MDA-MB-231-GFP spheroids cultured 14 days post P7C3 exposure. [B]. Quantitative analysis of spheroid size. ***p* < 0.01, *****p* < 0.0001.

**Supplementary Figure S15**

**
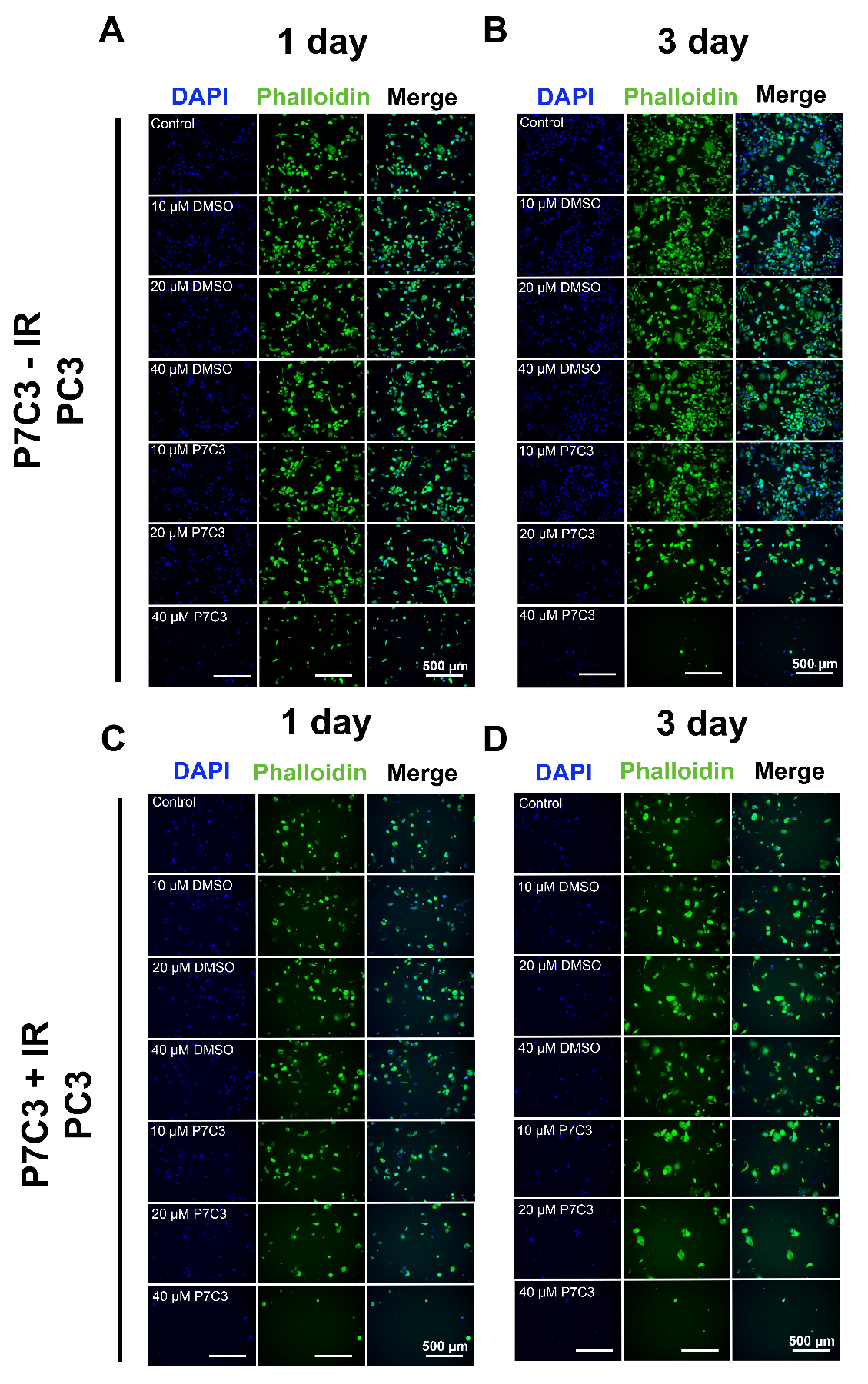
**

**Supplementary Figure S15. Exposure of PC3 cells to P7C3 ± IR decreases cell metabolic activity in a dose-dependent manner.** [A-D] Representative confocal micrographs of PC3 cells at 1 day and 3 days post P7C3 exposure. Cells were fixed and the nuclei and actin filaments were stained with DAPI (blue) and phalloidin (green), respectively, prior to examination using confocal laser scanning microscopy.

**Supplementary Figure S16**

**
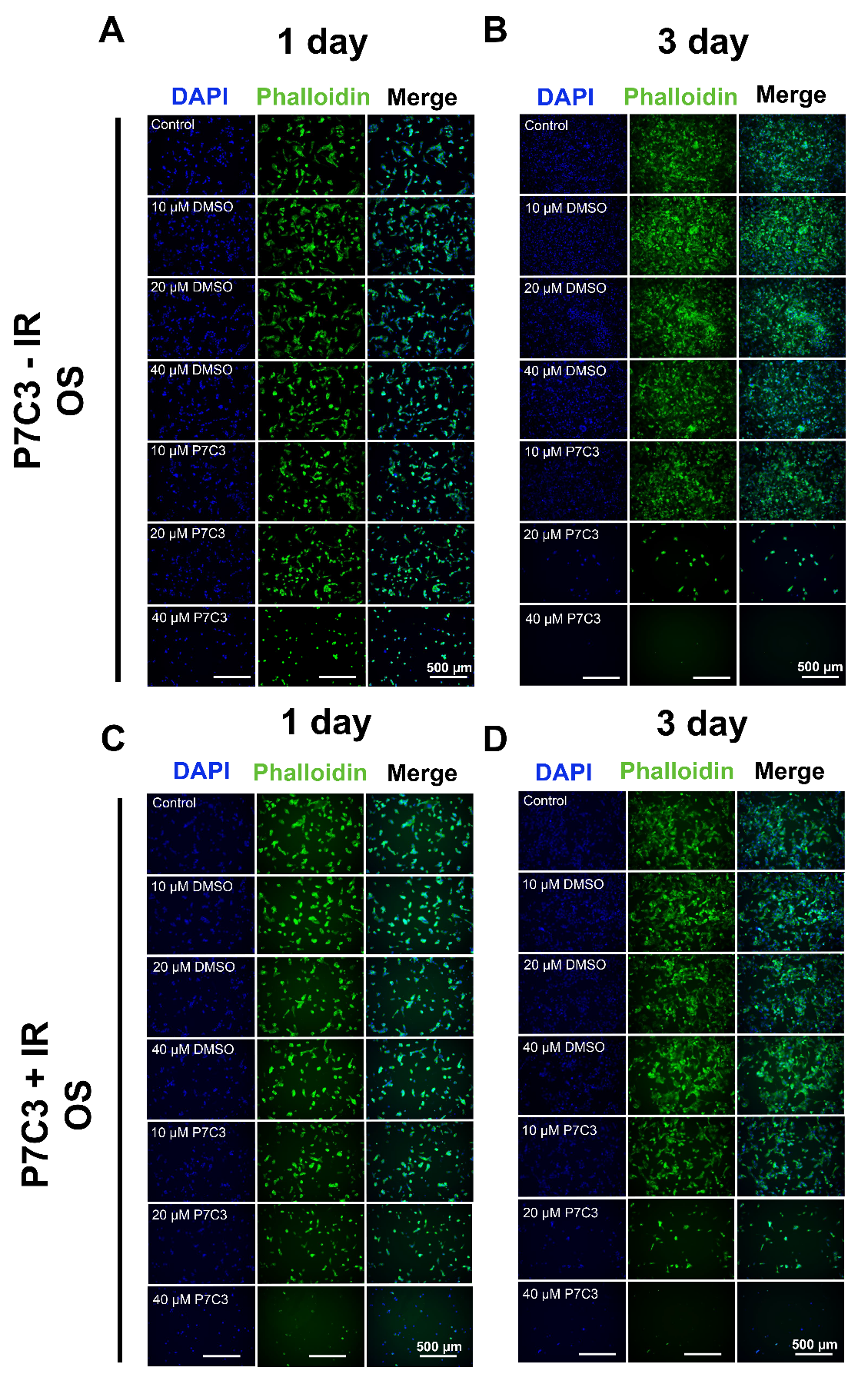
**

**Supplementary Figure S16. Exposure of human osteosarcoma cells to P7C3 ± IR decreases cell metabolic activity in a dose-dependent manner.** [A-D] Representative confocal micrographs of human osteosarcoma cells following incubation with increasing concentrations of P7C3 or solvent control for 1 day or 3 days. Cells were fixed and the nuclei and actin filaments were stained with DAPI (blue) and phalloidin (green), respectively, prior to examination using confocal laser scanning microscopy.

**Supplementary Figure S17**

**
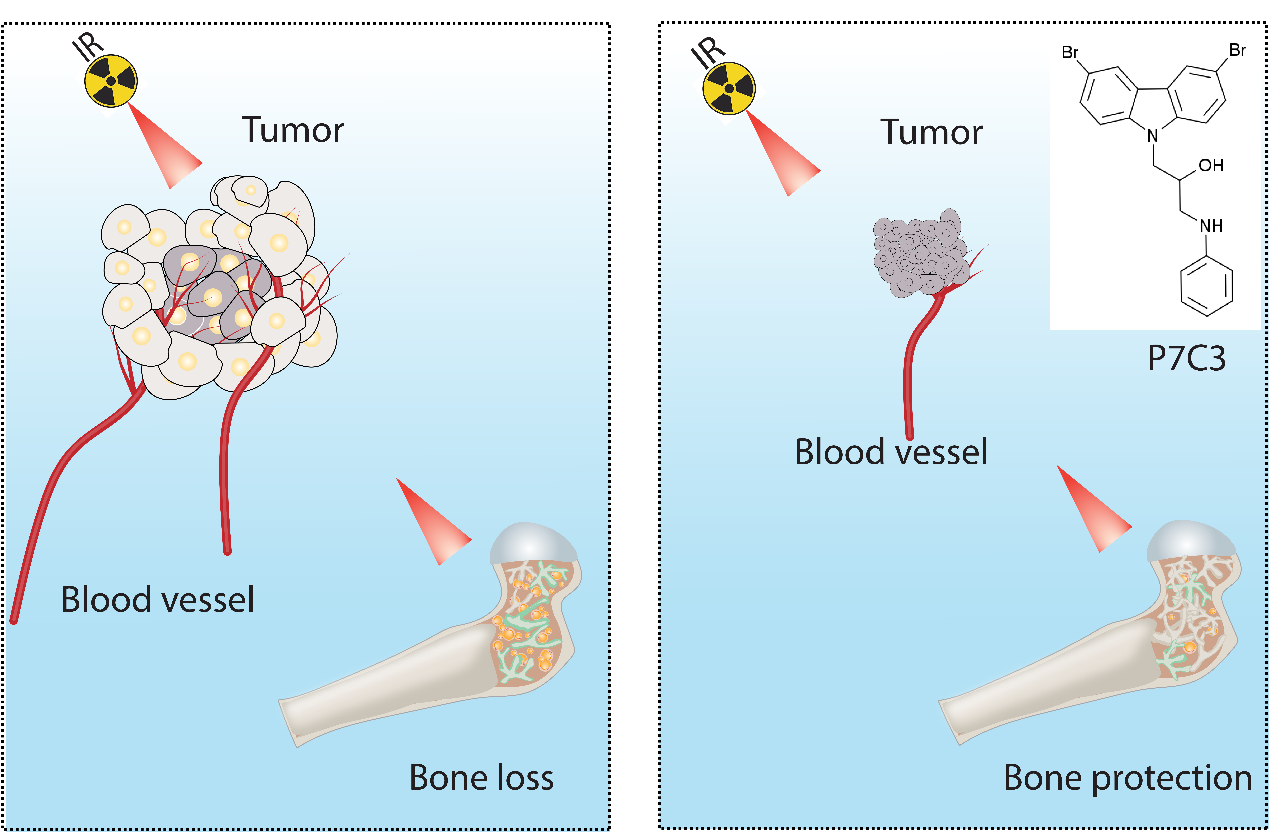
**

**Supplementary Figure S17. Schematic diagram highlighting the potential protective effect provided by P7C3 against irradiation-induced bone loss.** IR-induced non-cancerous tissue damage, especially to musculoskeletal tissue, is a significant cause of pain, where no effective therapeutic strategies exist. In this study, we examined the exogenous pre-treatment and daily administration of 20 mg/kg P7C3 during IR and *in vivo*. Results demonstrated a radioprotective effect against IR-induced bone damage, where P7C3 shifted the imbalance from osteoclastogenesis toward osteogenesis along with a significant reduction in IR-induced bone marrow adiposity and senescence. The results presented in this study hold translational promise for preventing IR-induced bone loss.
